# Electron Transport and Nonlinear Optical Properties of Substituted Aryldimesityl Boranes: A DFT Study

**DOI:** 10.1371/journal.pone.0114125

**Published:** 2014-12-05

**Authors:** Altaf Hussain Pandith, Nasarul Islam

**Affiliations:** Department of Chemistry, University of Kashmir, Srinagar, Kashmir, India; Gazi University, Turkey

## Abstract

A comprehensive theoretical study was carried out on a series of aryldimesityl borane (DMB) derivatives using Density Functional theory. Optimized geometries and electronic parameters like electron affinity, reorganization energy, frontiers molecular contours, polarizability and hyperpolarizability have been calculated by employing B3PW91/6-311++G (d, p) level of theory. Our results show that the Hammett function and geometrical parameters correlates well with the reorganization energies and hyperpolarizability for the series of DMB derivatives studied in this work. The orbital energy study reveals that the electron releasing substituents increase the LUMO energies and electron withdrawing substituents decrease the LUMO energies, reflecting the electron transport character of aryldimesityl borane derivatives. From frontier molecular orbitals diagram it is evident that mesityl rings act as the donor, while the phenylene and Boron atom appear as acceptors in these systems. The calculated hyperpolarizability of secondary amine derivative of DMB is 40 times higher than DMB (**1**). The electronic excitation contributions to the hyperpolarizability studied by using TDDFT calculation shows that hyperpolarizability correlates well with dipole moment in ground and excited state and excitation energy in terms of the two-level model. Thus the results of these calculations can be helpful in designing the DMB derivatives for efficient electron transport and nonlinear optical material by appropriate substitution with electron releasing or withdrawing substituents on phenyl ring of DMB system.

## Introduction

A great deal of interest has recently been aroused in organic materials for their active role in opto-electronic devices such as photovoltaic cells and organic light-emitting diodes (OLEDs) [1]. The practically useful OLEDs require good luminescence, efficient charge transport and fast energy transfer properties. The simplest OLED consist of anode, an electron transport material (ETM) layer, a hole transport material (HTM) layer and a cathode. Organic material used as ETM lag behind due to obstacles such as scarce solubility, difficult processibility, and instability in air [2]. Thus, one of the key challenges in developing OLEDs is the design of stable ETM with high efficiency of electron ejection and adequate electron mobility.

The photochemical investigation of organoboron compounds reveals that triphenyl boranes in presence of donor solvents undergo photodegradation, in contrast to trimesityl boranes, which were found photochemically inert in all these solvents. It was observed that two mesityl groups were significant in providing an extra degree of stability, which attracted considerable research interest in carrying out photophysical studies of p-substituted aryldimesityl boranes (DMB) [2]–[6]. The three coordinated boron is electron deficient with its vacant **pz** orbital. Due to strong π-acceptor character, a significant delocalization occurs when conjugated with an adjacent organic π-system. The acceptor character of the dimesitylboryl group was found similar to cyano group than the nitro group. But unlike other **π-**acceptors, it is a weak σ-electron acceptor as electronegativity of boron is less than that of carbon. Thus, when boron bonded to organic systems it may even function as a σ-electron donor [4]–[9]. The presence of vacant **pz** orbital make boron compounds prone to nucleophiles, resulting in either bond cleavage or the formation of a four-coordinate borate species that causes interruption in conjugation with adjacent **π**-systems. However, the organic system like mesityl (2,4,6- trimethylphenyl), forms a propeller like structure around the borane, and a cage is formed by methyl groups on aryl ring around the vacant **pz**-orbital [3]–[7], which enhances inertness of the borane compound. It has been found that these kinds of materials are applicable in a wide range of fields, such as nonlinear optics, single and biphotonic absorption materials, electron transporting and emissive materials in organic light emitting devices as well as chemosensors [7]–[21]. Shirota and others have reported several three-coordinate organoboron compounds as electron transport/hole-blocking materials for OLEDs [21]–[27].

The development of high-performance charge transport materials is a key issue for the fabrication of high-performance organic light emitting devices. The charge transport in molecular organic materials can be viewed in light of two theories, the band theory [28] and the hopping model [29]. In the band theory, transport of charge is an activationless process occurring through bands formed by the overlapping MOs between neighboring molecules. However in the hopping model charge transfer occurs between small coupled neighboring molecules. According to Marcus electron-transfer theory the rate of electron or hole transfer *k_et_* is given by following equation [30], [31]

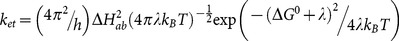
(1)


Where λ and ΔH_ab_ are the reorganization energy for the intramolecular electron transfer and the electronic coupling integral between donor-acceptor pair, respectively, and ΔG^0^is the Gibbs free energy change of the process. Efforts for several years in theoretical investigations of charge transport properties for several OLED materials confirm that both the reorganization energies (λ) and electron coupling parameter (ΔH_ab_) play an important role in the electron/hole transport process [32], [33] and can be evaluated by Quantum chemical calculations. The most well-known electron transport material used in OLEDs is Alq3, in which three 8-hydroxyquinolato (q) ligands chelate to the Al^3+^ ion in an octahedral environment. Tris(8-hydroxyquinolinato)aluminum (Alq3) has been widely used in organic light-emitting diodes (OLEDs)both as electron transport and light-emitting materials. According to the Marcus theory electron transport mainly depends on electronic coupling between donor and acceptor and the reorganization parameters for the two states upon electron transfer. Lin *et al*, calculated the reorganization energies of Alq3 for hole and electron transport. However, the slightly smaller value of reorganization energy for hole transport (0.242 eV) as compared to reorganization energy for electron transport (0.276 eV) suggest that Alq3 posses better hole transport than electron transport property, apparently contradicting the experimental observations [34]. Therefore, it was concluded that the electronic coupling factor (Δ*H*_ab_)may be a dominant factor in influencing charge transport in a system [35]. The first-principle band-structure calculations using density-functional theory with generalized gradient approximation by Yang *et al*., reveals that the inter band gaps within the unoccupied bands are generally smaller than those for the occupied bands, which indicate that the electron can hop from one band to another much easier than the hole, through electron-phonon coupling for instance, therefore, explaining the larger mobility for the electron than for the hole [36]. In the present work, we have adopted quantum chemical calculations to analyze several substituted DMB derivatives, at electronic structure level, for their potential character as electron transport materials. The choice of substituents has been made in such a way that they range from strong electron releasing groups (-(CH_3_)_2_N) to strong electron withdrawing ones (-NO_2_). Our investigation on boron compounds is motivated by the scope of this material as potential conducting material having application in OLEDs.

Photo-physical properties of DMB make them suitable candidates having potential use in the design and development of materials exhibiting large second-order nonlinear optical (NLO) properties. NLO response of molecular system is governed by the parameters depending on the extend of the conjugation and the strength of substituent groups in terms of their donor or acceptor nature [37]. Earlier studies of the structure–property relationships of such molecules indicate that the hyperpolarizability (β) of the molecule changes with a change in donor and acceptor strength and with increase in the extent of π- conjugation [38], [39]. At molecular level, nonlinear optical susceptibilities are determined by the first and second hyperpolarizabilities. The present work is intended for the optimization of the molecular structure-NLO properties relationships of DMB with conventional electron donor or acceptor substituent groups on the phenyl ring. Investigations have been made to study the substitution effect in the DMB derivatives and its influence on their energy levels, nonlinear optics and electron-transport properties. In particular, the molecular geometries, HOMO and LUMO levels, ionization potential, electron affinity, reorganization energy, and molecular orbitals shapes have been investigated as they are directly related to such OLED's characteristics like efficiency and durability.

### Theoretical Calculations

In this work all theoretical calculations were carried out at the density functional theory (DFT) level of theory, performed by using Gaussian 03 computational package [40]. Geometry optimizations were performed on isolated entities in gaseous phase, employing Becke's 3 Perdew Wang 91 (B3PW91) [41] exchange-correlation functional using 6-311++G (d, p) basis set [42]. All the geometries were characterized as minima with zero imaginary frequencies. The neutral molecules were treated as closed-shell systems; while for the radical anion open-shell system optimizations were carried out using a spin unrestricted wave function (UB3PW91 procedure). Vertical electronic excitation energies were computed by using the time-dependent density functional level of theory (TD-DFT) [43]. The TDDFT/B3PW91 level of theory has been found quite efficient, for performing significantly single-excitation calculations for the low-lying valence excited states of both closed shell and open-shell molecules [44]. The static polarizability and hyperpolarizabilities were calculated using derivative method which relates different derivatives of energy or dipole moment to various coefficients of power series expansions. The total static dipole moment (μ), average linear polarizability (α) and first-order hyperpolarizability (β) using the x, y, z components from Gaussian 03 W [40] output were calculated by the equation 2, 3 and 4 [45]–[47].
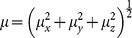
(2)

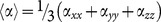
(3)

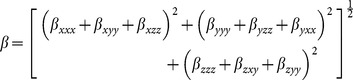
(4)


## Results and Discussion

**Figure 1:**
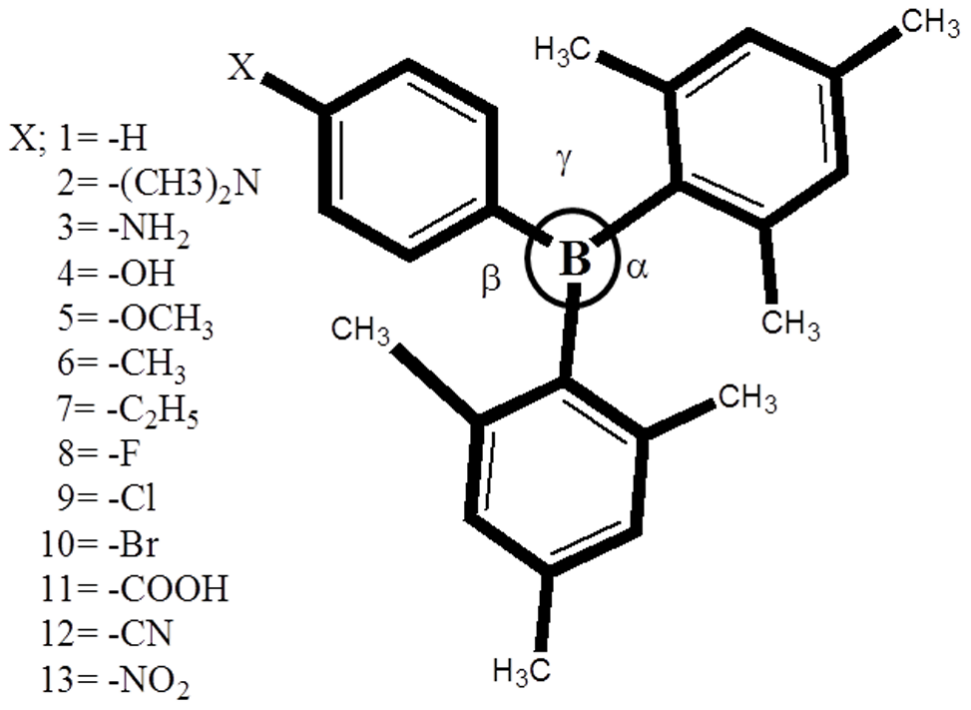
Sketch of aryldimesityl borane (DMB) derivatives study using DFT at B3PW91/6-311++G (d,p)level of theory.

*Ab initio* calculations were carried out on a series of DMB monomers with donor (**2–7**) and acceptor (**8–13**) substituents as shown in Figure 1. The substituents were chosen on the basis of their ability to affect electron density of the phenyl ring, covering possibly wide range of the Hammett parameter (σ) [48] from strong electron releasing groups to strong electron withdrawing ones. According to Hammett equation, the electron releasing substituents have negative and the electron withdrawing groups have positive value of Hammett parameter. In this work, we present a comparative effect of substitution by correlating theoretically calculated values of interest with Hammett parameter and the analysis of the observed relationships. In the first section, we have studied the electronic properties and their correlation with Hammett parameter and geometrical parameters. In second section, we have studied the nonlinear optical properties of DMB in terms of polarizability and hyperpolarizability for all systems. The calculations have been carried out for neutral molecules, radical-anion as well as radical-cation.

### Optimized Geometry

The optimized geometrical structures of the studied systems **1–13** are shown in Figure S1, the coordinates of all optimized DMB derivatives are given in Table S1 and some selected bond lengths, bond angles of parent DMB are listed in Table S2. We found a reasonable agreement between the calculated geometrical parameters (bond lengths and bond angles) and the experimental values obtained from the X-ray diffraction data [49] for the DMB(**1**) molecules (Table-S2). The slight deviations observed may be attributed to the fact that theoretical calculations are done for single isolated molecule in gaseous phase and the experimental values are those of the crystalline solid phase. This indicates that the adopted density functional and basis set are feasible for the studied systems. The un-substituted DMB(**1**) is composed of two mesityl rings and a phenyl ring bonded to a central boron atom. The central BC_3_ moiety is planar and forms an equilateral triangle. The two mesityl and phenyl rings are twisted from that plane so the whole molecule can be visualized as a slightly distorted propeller. The C-B bond lengths for two mesityl rings are equal to1.570 Å, and C-B length for phenyl ring is 1.581 Å and the torsion angle of the phenyl ring with the mesityl amounts to about 21.52^0^ in the neutral state. The optimized geometric parameters of DMB(**1**) calculated in this study are presented in Table 1. We studied in detail different derivatives of DMB, choosing carefully different functional groups in varying order of their electron releasing (N(CH_3_)_2_, NH_2_, OH, OCH_3_, C_2_H_5_, CH_3_) and electron withdrawing(F, Cl, Br, COOH, CN, NO_2_) character at para-position (phenyl) of DMB, as shown in Figure 1. From the optimized geometries, we found that upon substitution of hydrogen with electron releasing groups, C_18_-B bond length increase as [**2**<**3**<**4** = **5**<**6** = **7**] compared to parent molecule (**1**), and dihedral angle (<CBCC) increase in the order [**2**(17.26^0^) <**3**(18.12^0^) <**5**(21.02^0^) <**4**(19.01^0^) <**6**(21.07^0^) <**7**(21.31^0^)] compared to 21.52^0^ angle in parent molecules. In electron withdrawing derivatives the bond length does not show a significant change as a function of electron withdrawing character of the substituents. However, in these derivatives the dihedral angle (<CBCC) is greater than the parent molecule and increase in the order [**8**(20.66^0^) <**9**(21.62^0^) <**10**(22.17^0^) <**11**(22.98^0^) <**12**(24.01^0^) <**13**(24.65^0^)]. The effect of substitution on the aromaticity was analyzed by bond length alternation (BLA). We calculated a local BLA associated with the C_23_-C_20_, C_18_-B, C_20_ = C_18_ lengths according to the definition given by Fu *et al*. [50]. Thus, the BLA is calculated by subtracting the arithmetic mean of the two central C-C bonds and the central C = C bond length

**Table 1:** Selected geometrical parameters (bond angles and bond lengths) of aryldimesityl borane (DMB) derivatives in neutral and anionic states calculated at B3PW91/6-311++G (d, p) level.

Derivative	Bond angle (deg.) <C_2_-B-C_10_		Bond angle (deg.) <C_2_-B-C_18_		Bond length (Å)B-C_18_		Bond length(Å)B-C_10_		Dihedral angle (deg.) <C-B-C-C	
	Neutral	Anionic	Neutral	Anionic	Neutral	Anionic	Neutral	Anionic	Neutral	Anionic
1	122.172	120.908	118.813	119.849	1.581	1.594	1.570	1.553	21.52	23.41
2	121.502	120.602	119.247	119.673	1.587	1.591	1.551	1.554	17.26	19.21
3	121.658	120.718	119.155	119.643	1.586	1.591	1.554	1.559	18.12	19.34
4	122.047	120.734	119.002	119.667	1.583	1.591	1.560	1.552	19.01	19.64
5	122.028	120.667	119.053	119.629	1.583	1.591	1.560	1.557	21.02	22.57
6	122.224	119.839	118.898	120.144	1.582	1.597	1.566	1.548	21.07	22.95
7	122.474	120.225	118.732	119.848	1.582	1.594	1.567	1.552	21.31	23.21
8	122.460	120.757	118.780	119.902	1.580	1.604	1.567	1.601	20.66	21.57
9	122.653	120.315	118.674	119.844	1.580	1.595	1.571	1.548	21.62	24.58
10	122.790	120.280	118.606	119.859	1.579	1.595	1.579	1.547	22.17	27.57
11	122.880	119.530	118.640	120.250	1.577	1.600	1.577	1.537	22.98	31.09
12	123.115	119.704	118.442	120.149	1.577	1.600	1.578	1.532	23.01	32.17
13	123.227	119.667	118.387	120.167	1.576	1.600	1.580	1.529	23.04	33.54



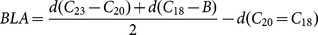
(5)


The increase in electron density due to electron donating groups on parent DMB show an increased double bond character of C_23_-C_20_ and C_18_-B bonds and decreased double bond character of C_20_ = C_18_ bond in the order of 7<6<5<4<3<2. The BLA values increases from 0.056 (**2**) <0.059(**3**) <0.061(**4**)  = 0.061(**5**) <0.063(**6**) <0.064(**7**), indicating that the degree of conjugation is increasing upon the substitution of the parent DMB with electron donating groups. However, there is no good correlation between BLA and the electron withdrawing capacity of substituents. The DMB(**12**) and DMB(**13**) have maximum electron withdrawing capacity, with highest BLA values 0.083 and 0.085 respectively, but in case of **8**, **9**,**10** electron withdrawing capacity decreases as **8**>**9**>**10**, but the BLA values does not differ much (0.070(**8**)  = 0.070(**9**) <0.071(10).

### Electron Transport Properties

**Figure 2:**
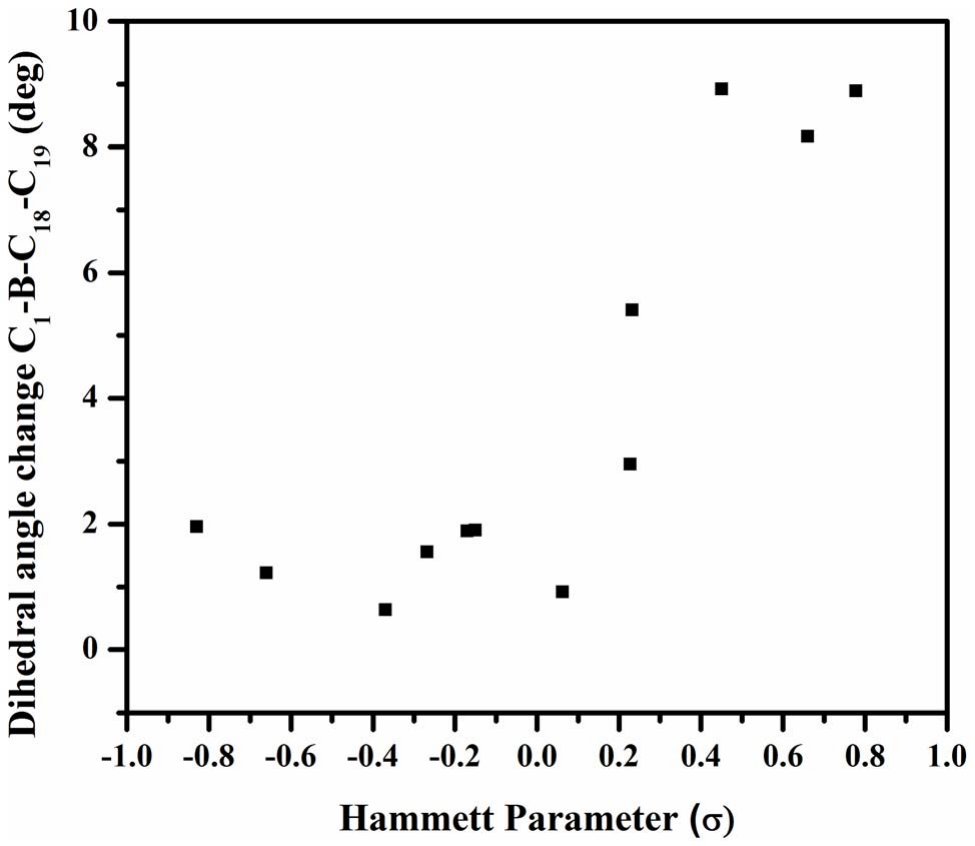
Plot of geometrical change (dihedral angle) with the Hammett Parameter, for the series of studied DMB derivates.

To study electron transport property of DMB systems, we optimized the anion geometry to understand the effects of charge injection on the molecular conformational stability, in terms of structural distortions and changes in electronic structure, by varying the nature of substituents. The calculations show that neutral molecules show an overall negligible change in geometrical parameters upon the formation of radical anion in all the derivatives (Table 1). There is a change in dihedral angle <CBCC between the mesityl ring and phenyl ring from 22.15^0^ to 24.05^0^ and the bond length of C_18_-B bond slightly changes from 1.581 Å to 1.594 Å in case of DMB(**1**). The structural reorganization from neutral to anionic species, reflected by the change in C_18_-B bond length upon electron injection, shows the following trend; (-N(CH_3_)_2_(**2**) <-NH_2_(**3**) <-OH(**5**) <-OCH_3_(**4**) <-C_2_H_5_(**6**) <-CH_3_(**7**) <-F(**8**) <-Cl(**9**) <-Br(**10**) <-COOH (**11**) <-CN(**12**) <-NO_2_ (**13**) [0.004 Å <0.005 Å <0.007 Å <0.008 Å<0.015 Å>0.012Å = 0.012 Å <0.15 Å <0.16 Å <0.23 Å  = 0.23 Å <0.24 Å], respectively. This data reflects that the contribution to activation barrier from geometrical reorganization for electron transport is small in case of electron releasing derivatives as compared to electron withdrawing derivatives. The magnitude of structural modifications increases on moving from the electron releasing substituents to the electron withdrawing substituents. This indicate the existence of strong correlation between Hammett parameter (σ) and the geometrical parameters, as is evident from the plots of geometrical parameters verus σ, shown in Figure 2 and Figure S2. The enhanced change in dihedral angle due to electron withdrawing groups represents the greater tendency of these molecules to undergo structural change upon radical-anion formation from their neutral states, as compared to molecules with electron releasing groups. The main influence of the injection of one negative charge is manifested in the increase in bond length of B-C_18_ substituted DMBs radical anion, which may be responsible for decrease in its rigidity. However, we found that, in general, the optimized anionic geometries show small structural changes comparative to the neutral molecules, the structural changes being close to Alq_3_ [35]. Thus, the injection of one negative charge does not affect the structural stability of DMBs derivatives and this property may allow tuning them into better electron transport systems.

**Figure 3:**
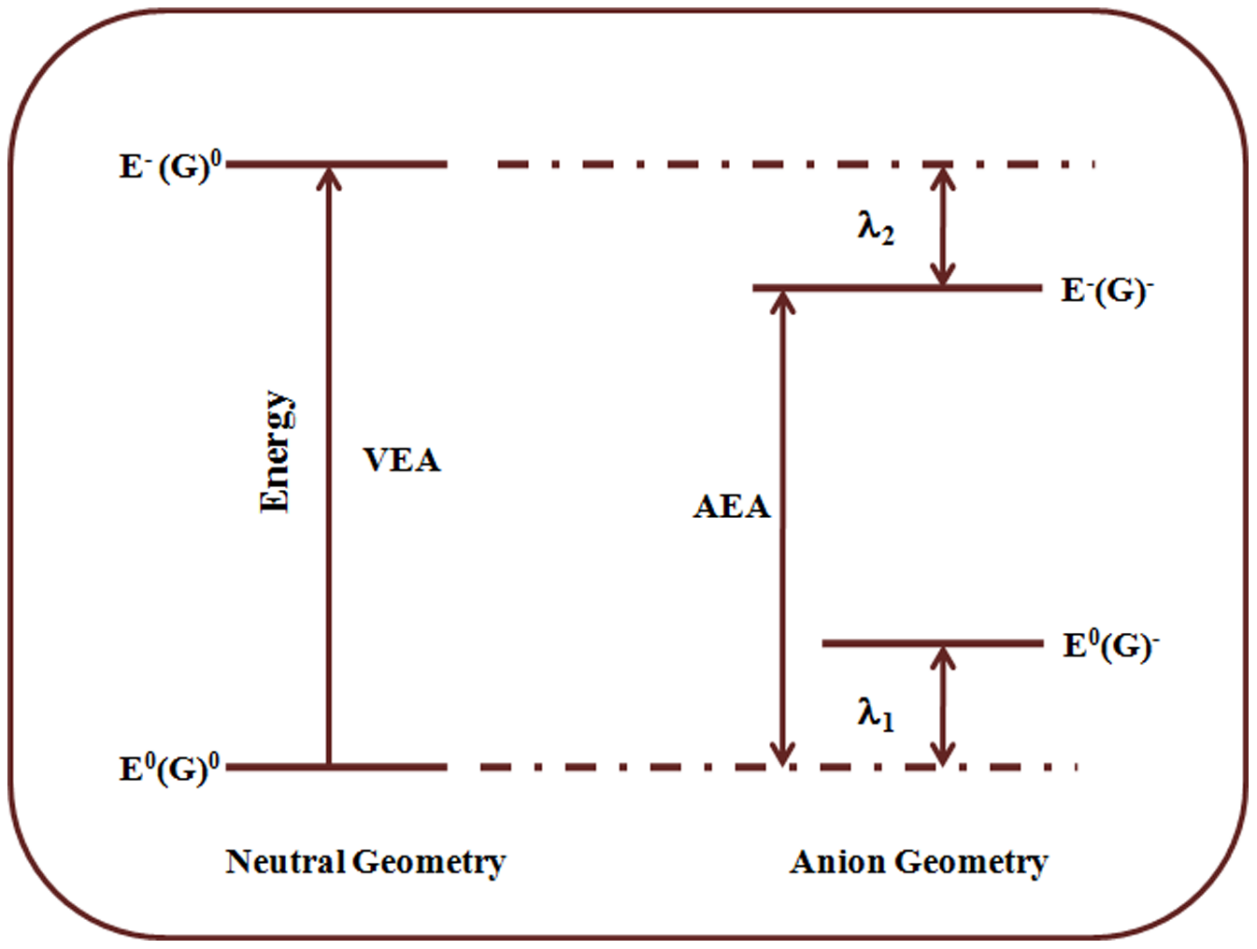
Calculation details of the reorganization energy (eV) for the electron transport, λ_1_ is reorganization energy of a neutral molecule and λ_2_ reorganization energy of a anionic-radical.

We calculated electron affinities EA, ionization potential (IP) and reorganization energies (λ_1_, λ_2_, and λ_i_) for electron transport using the Figure 3. According to Marcus Model, the rate of intramolecular electron transfer depends mainly on the reorganization energy and the electronic coupling between the donor-acceptor pair, in addition to the overall exergonicity of the process. For a self-exchange electron transfer process, the Gibbs free energy change (ΔG) is zero and the rate of electron transfer only depends on intrinsic activation barrier, signified by (inner and outer) reorganization energy (λ), and the electronic coupling parameter(ΔH_ab_). The inner intramolecular reorganization energy (λ_i_) for self-exchange process has two contributions, arising from the geometry relaxation along inter-nuclear coordinate upon moving from neutral-state geometry to the charged-state geometry and vice versa;
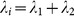
(6)


λ_1_ and λ_2_ can be calculated directly from the adiabatic potential energy surface as;
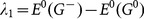
(7)

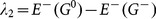
(8)


Where **E^0^(G^0^)** and **E^-^(G^-^)** are the ground-state energies of the neutral and anionic states, respectively, and **E^0^(G^-^)** and **E^-^(G^0^)** are the energy of the neutral molecule at the optimal anionic geometry and the energy of the anionic molecule at the optimal geometry of the neutral molecule.

**Figure 4:**
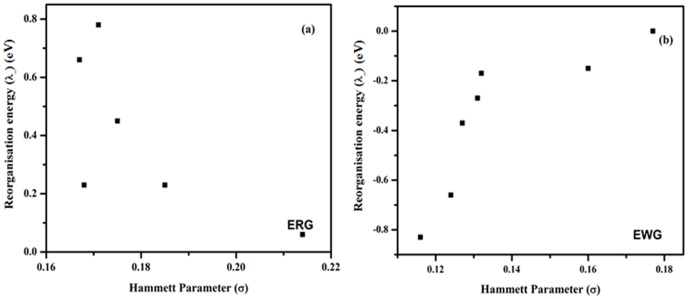
Plot of reorganization energy (eV) with Hammett parameter of (a) DMB derivatives with electron releasing substituents, (b) DMB derivatives with electron withdrawing substituents.

The calculated reorganization energies for electron transport **λ_1_**, **λ_2_** and **λ_i_** are shown in Table 2 From Table 2, it is clear that the value of λ_i_ decrease both for DMB derivatives with electron releasing (**2–7**) and electron withdrawing (**7–13**) substituents as compared to non-substituted DMB (**1**), with exception of compound **8** and **9**, where it slightly increases. The calculated values of inner sphere reorganization parameter (λ_i_)for different derivatives largely correlates well with the order of structural changes from neutral to anionic species, reflected by the change in C_18_-B bond length, upon electron injection. The order of change in reorganization energy is much more uniform in case of electron releasing substituents, as compared to electron withdrawing substituents. In case of electron withdrawing group the reorganization energies follow the trend: _CN <_NO_2_<_Br <_COOH <_Cl <_F, with maximum for fluorine (0.214 eV) derivative which is, however, lower than to Alq_3_ (0.276 eV) [35], [51]. The smaller reorganisation energy value of DMB derivatives as compared Alq_3_ will benefit the charge carrier transport. For the DMB with the electron releasing group, the reorganization energies has the following order: _CH_3_> _C_2_H_5_>_OH>_OCH_3_>_NH_2_> _N(CH_3_)_2_. The Figure 4 shows a correlation of reorganization energies **λ** with Hammett parameter. In case of electron withdrawing groups the trend is irregular, floro shows large geometrical changes as compared to strong electron withdrawing substituents, such as _NO_2_, _COOH, and _CN comparable to those of un-substituted DMB(**1**), whereas electron releasing substituents show clear trend; the stronger (_NH_2_<_N(CH_3_)_2_) cause the DMB derivatives to undergo lesser geometry changes compared to weak electron releasing substituents.

**Table 2:** Reorganization energy (λ_-_) for electron transport (eV) calculated by using DFT at B3PW91/6-311++G (d, p) level of theory and Hammett value (σ) of DMB derivatives.

Derivative	VEA(eV)	AEA(eV)	λ_1_	λ_2_	λ_-_ = λ_1_+λ_2_	σ*
1	0.702	0.612	0.087	0.090	0.177	0
2	0.378	0.326	0.064	0.052	0.116	−0.83
3	0.408	0.345	0.06	0.063	0.124	−0.66
4	0.556	0.487	0.058	0.069	0.127	−0.37
5	0.550	0.482	0.063	0.068	0.131	−0.27
6	0.608	0.536	0.060	0.072	0.132	−0.17
7	0.676	0.598	0.081	0.078	0.160	−0.15
8	0.712	0.599	0.101	0.113	0.214	0.06
9	0.876	0.787	0.096	0.089	0.185	0.23
10	0.913	0.825	0.080	0.088	0.168	0.23
11	0.955	0.864	0.084	0.091	0.175	0.45
12	0.967	0.886	0.086	0.081	0.167	0.66
13	0.828	0.741	0.084	0.087	0.171	0.78

*reference [48]

Another important factor which characterizes the electron transport properties of such materials is their electron affinity. In an electron transport material, the electron affinity must be high enough to allow an efficient injection of electrons into low-lying unoccupied molecular orbitals. We calculated vertical and adiabatic electron affinities by using the equations 9 and 10 at B3PW91/6-311G (d,p)++ level of theory and the results are shown in Table 2.



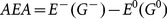
(9)

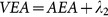
(10)


The theoretically calculated AEA for the molecular oxygen at the B3LYP/6-31+G* is 0.59 eV [52], while the experimental value, determined by photoelectron spectroscopy, is reported as 0.448±0.006 eV [53]. Except compounds (**2–5**), EAs calculated for the whole set of derivatives are higher than the values reported for the molecular oxygen. So, these compounds in anionic form seem to be quite stable towards quenching caused by molecular oxygen present in the atmosphere.

**Figure 5:**
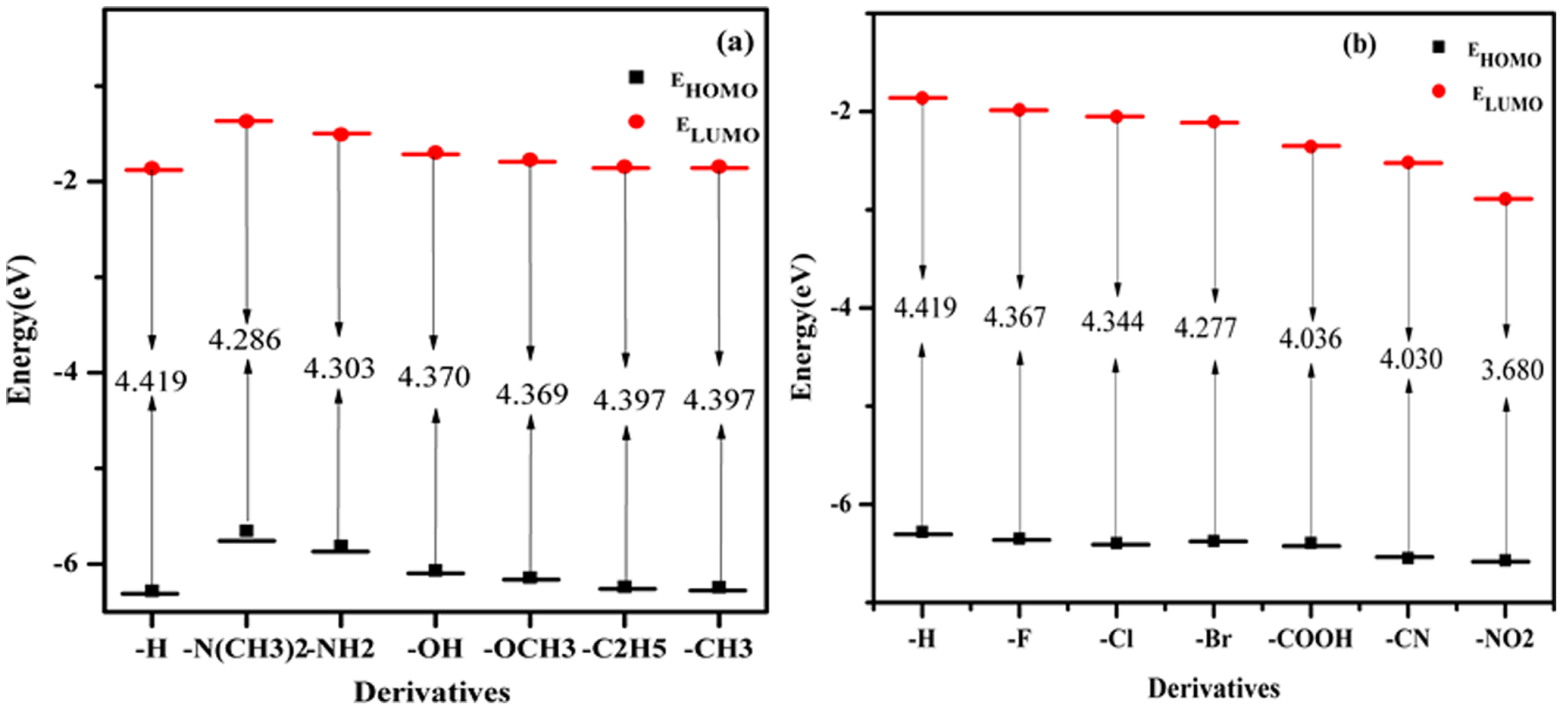
Calculated HOMO energies, LUMO energies of DMB derivatives (a) electron releasing substituents, (b) electron withdrawing substituents, together with the LUMO-HOMO gap at DFT/B3PW91/6-311++G (d,p) level of theory.

**Figure 6:**
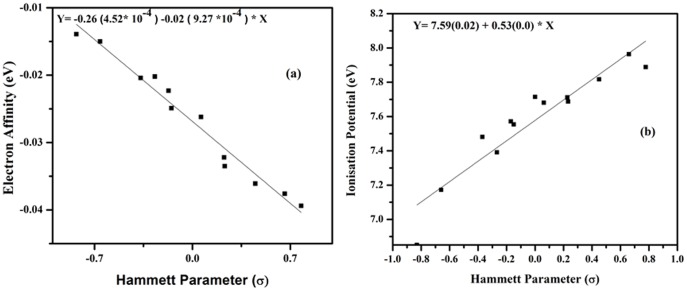
Plot of Hammett Parameter with (a) electron affinity (eV) and (b) ionization potential (eV).

**Figure 7:**
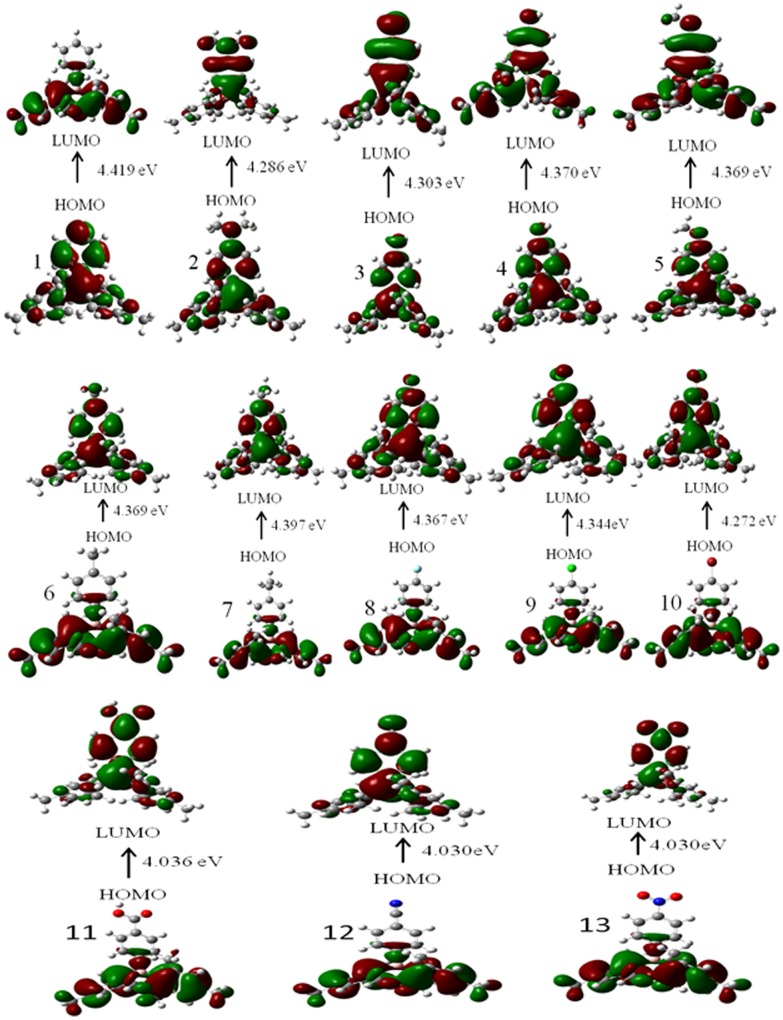
The frontier molecular orbitals (FMOs) of DMB derivatives at DFT/B3PW91/6-311G (d,p)++ level of theory.

Electrical properties of molecular systems depend on the energies of levels allowed for electrons/holes and energy gap between these levels. The energies of the HOMO and LUMO levels used for the calculation of the energy gap were calculated using DFT employing B3PW91/6-311++G(d, p) level performed for isolated molecules (Table 3). It is clear from Table 3 that the electron releasing substituents(**2–7**) increase the LUMO energy of the DMB with increasing order of their electron donating behavior and, therefore, electron affinity of these DMB derivatives decreases in that order (Figure 5(a)). Whereas, the electron withdrawing groups (**8–13**) decrease the LUMO energy of the DMB with increasing order of their electron withdrawing character and, as a consequence, electron affinity of these DMB derivatives increases with increase in the electron withdrawing character of the substituents (Figure 5(b)). A similar trend is observed for the HOMO energies and ionization potential. Simply this behavior is explained by build of enhanced effect of π-conjugation due to electron-releasing environment created by electron rich substituents and decrease of electron densities by electron withdrawing substituents. However, it is observed that with electron releasing groups as substituents (**2–7**), the changes in the HOMO energies are larger than those of LUMO energies, whereas with electron withdrawing groups as substituents, the changes in LUMO energies are greater than HOMO energies. One important observation is that the HOMO-LUMO gap, ΔE(H-L), of the studied compounds decrease as compared to un-substituted DMB and this gap goes on decreasing with increase in the electron donating as well as electron withdrawing character of the substituents. Therefore, it is possible to tailor the HOMO-LUMO gap in such electron transport materials by rational variation of substitutions on the basis their position on the Hammett parameter (σ) scale. It was not possible to compare the calculated IP and EA with the experimental values due to lack of experimental data, however the plot of IP and EA correlated quite well with σ, which points out an obvious substitution effect on energy levels in the studied systems (Figure 6). The frontier molecular orbitals (FMOs) of all the systems are shown in Figure 7. It was observed that the highest occupied molecular orbitals (HOMOs) of the neutral molecules delocalize primarily over the two mesityl rings, whereas the lowest unoccupied molecular orbitals (LUMOs) localize largely on the Boron (B) atom and the phenylene of the DMB moiety, with a small contribution from electron releasing or withdrawing substituent. Thus, mesityl rings constitute the donor state, while the phenylene and B atom as the acceptors state in these systems. Compared to Alq3 [51] the LUMO is more delocalized than HOMO in case of DMB derivatives, indicating that the electron can easily move among the DMB molecule which will facilities enhanced electron transportation, particular in case of DMB derivatives with electron donating substituents. The HOMO-LUMO electronic transitions can be attributed to charge transfer from the two mesityl rings to the phenylene and the vacant p_z_ of Boron atom. Thus, the minimization of the barrier for electron injection and transport can be achieved by proper adjustment of EA of given derivative.

**Table 3:** DFT calculated HOMO, LUMO energies, Ionisation potentials (Ip) and Electron affinity (EA) of studied aryldimesityl borane(DMB) derivative calculated.

Derivative	E_HOMO_ (eV)	E_LUMO_ (eV)	ΔE_HOMO-LUMO_ (eV)	aΔ(HOMO) (eV)	bΔ(LUMO) (eV)	cIp (eV)	^d^EA (eV)
1	−6.281	−1.862	4.419			7.714	0.702
2	−5.655	−1.369	4.286	0.625	0.493	6.851	0.378
3	−5.812	−1.509	4.303	0.469	0.353	7.172	0.408
4	−6.066	−1.696	4.370	0.215	0.166	7.481	0.556
5	−6.141	−1.773	4.369	0.139	0.090	7.391	0.550
6	−6.240	−1.843	4.397	0.041	0.019	7.571	0.608
7	−6.243	−1.846	4.397	0.038	0.017	7.554	0.676
8	−6.349	−1.982	4.367	−0.068	−0.119	7.680	0.712
9	−6.395	−2.052	4.344	−0.115	−0.189	7.711	0.876
10	−6.374	−2.102	4.272	−0.093	−0.240	7.687	0.913
11	−6.393	−2.357	4.036	−0.112	−0.495	7.817	0.955
12	−6.547	−2.517	4.030	−0.266	−0.654	7.964	0.967
13	−6.570	−2.889	3.680	−0.289	−1.027	7.888	0.828

a Δ(HOMO)  = E(_HOMO DMB derivative_) – E(HOMO_DMB_),

b Δ(LUMO)  = E(_LUMO DMB derivative_) – E(HOMO_DMB_),

c Ip = E^+^(G)^0^- E^0^(G)^0^ and ^d^EA(eV)  = E^-^(G)^0^- E^0^(G)^0^.

**Figure 8:**
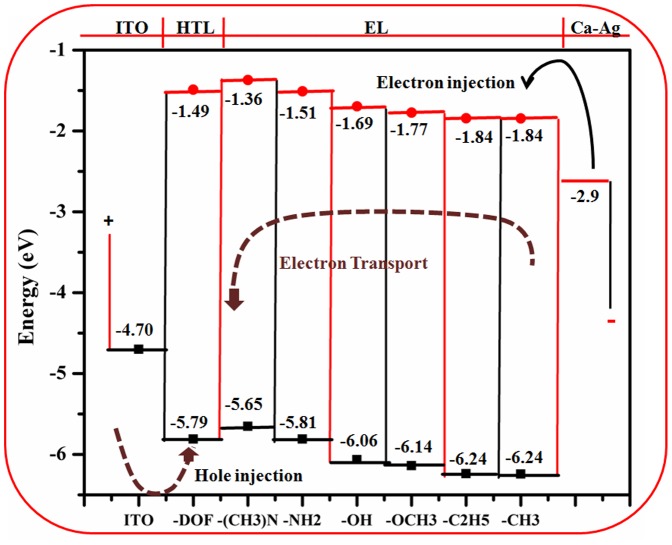
Schematic diagram of a two-layered OLED device.

The theoretical data obtained for electron transport of DMB derivatives can be used to model an OLED of desired properties. We used a single OLED based on ITO(Indium tin oxide) as an anode, 9,9-dioctyfluorene as a hole transport layer, Ca-Ag as cathode and some studied TBA derivatives as an electron transport/emitting layer(EL) (Figure 8). The first parameter that was considered in analysis was electron injection from Ca-Ag into EL. Efficiency of this process depends upon the energy barrier between the Ca-Ag work function and LUMO level of EL. Lower the barrier, the higher the injection efficiency. On comparing, we see that the DMB derivatives with electron donating substituents have lower energy barrier. Another parameter is efficiency of hole transfer from HTL to EL which is significant in case of derivatives **2** and **3**, having least energy barrier between HOMO of ITO and the derivatives.

### Nonlinear Response

According to Oudar and Chelma, first hyperpolarizability (β) is related to low-lying charge-transfer transition through a two level model [54] on the basis of the complex sum over states (SOS) expression as;
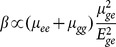
(11)where (μ_ee_-μ_gg_) is the difference between the dipole moments of the *m*th excited state and ground state respectively, μ_ge_ is the transition dipole, and E_ge_ is the transition energy.

In the two-level expression, the first hyperpolarizability (β), is proportional to the square of transition dipole and is inversely proportional to the square of the transition energy. For a molecule, the transition energy is therefore the decisive factor for large β values. According to two-level model materials with well-performing NLO property should possess a low lying Charge Transfer excited states with good oscillator strength [55]–[57]. The calculated values of β, μ_ge_ and E_ge_ employing TDDFT using B3LYP/6311++G (d, p) level of theory are given in Table 4. From the Table 4, it is clear that there is an enhancement of β values with increasing donor/acceptor abilities by substitutions as compared to DMB (**1**). Thus, on account of above equation the value of β increase with the increasing μ_ge_^2^ and decreasing E_ge_ values. The results show that in case of electron releasing groups the observed trend for μ_ge_^2^/E_ge_^2^ values is **2**>**3**>**4** = **5**>**7**>**6** and is in agreement with the hyperpolarizability values. On comparing the –OCH_3_ and –N(CH_3_)_2_ substituted molecules (**5** and **7**) with the methyl substituted molecule (**6**), it is found that compound **6** possess smaller μ_ge_^2^ value. Thus, the hyperpolarizability of the –N(CH_3_)_2_ substituted molecule is five times that of the methyl substituted molecules. Comparing the value of μ_ge_^2^/E_ge_^2^ of electron releasing groups with electron withdrawing groups, the former have higher values of μ_ge_^2^/E_ge_^2^ than the latter ones, in general. The value of μ_ge_^2^/E_ge_^2^ for methoxy substituted compounds is lower than the halogen derivatives (**8, 9, 10**), however the hyperpolarizability of the halogen substituted molecules is higher than the methoxy substituted molecules. The probable reason is large electronegativity of halogen substituents as compared to methoxy group result in higher dipole moment along the X-direction cause enhance in β value of halogen derivatives [53]. The transition energy of methoxy derivative (4.369 eV) was found to be higher than the halogen DMB derivatives. Thus, the contribution of the square form (μ_ge_^2^/E_ge_^2^) has major effect on hyperpolarizability in the two-level model. The values of β for the two extreme such as nitro (strongest electron withdrawing group) and dimethylamine (strong electron releasing group) are completely according to two model equation and depend upon the transition dipole and transition energy of the group. Comparing the bromo and carboxy derivatives of DMB, the former has higher value of μ_ge_^2^/E_ge_^2^ but the latter has higher value of polarizability. Thus, the value of polarizability is also effected by other parameters like size, electronegativity of substituents etc. The first electronic transition is primarily composed of the HOMO-LUMO transition. As the carboxy group is replaced by the bromo, the HOMO energy does not change significantly; however, the LUMO is lowered. The electron density of the HOMO orbital of carboxy derivative would favor the charge transfer to the LUMO orbital, confined toward the acceptor part of a molecule due to reduction in HOMO-LUMO gap. Similarly, on comparing the dimethylamine and amine derivates of DMB, it was observed that the HOMO of the dimethylamine-DMB rise and the electron density shift, resulting in intense charge transfer compared to amine derivative and gives high β value for dimethylamine DMB derivative. From this observation, we may conclude that the first transition energy is an important measure of the hyperpolarizability of such molecules.

**Table 4:** Results of TDDFT calculations at B3PW91/6-311++G (d, p) level of theory for the electron transitions of DMB systems.

Derivative	λ_max_ (nm)	Major contribution	f	E_ge_(eV)	μ_ge_^2^	μ_ge_^2^/E_ge_^2^	β_total_(10^−30^esu)
1	331.66	HOMO-> LUMO (95%)	0.093	4.419	1.041	0.053	687.001
2	313.54	HOMO-> LUMO (80%) H-3-> LUMO (6%)	0.499	4.286	5.580	0.304	26889.281
3	316.50	HOMO-> LUMO (70%) H-2-> LUMO (4%)	0.293	4.303	3.150	0.170	15924.698
4	321.35	HOMO-> LUMO (89%) HOMO-> LUMO (7%)	0.091	4.370	1.009	0.053	8321.161
5	320.96	HMO-> LUMO (93%) HOMO-> LUMO (2%)	0.093	4.369	1.035	0.053	10813.668
6	328.56	HOMO-> LUMO (95%)	0.091	4.396	1.004	0.052	3156.490
7	328.20	HOMO-> LUMO (95%)	0.093	4.397	1.009	0.054	2512.611
8	331.26	HOMO-> LUMO (95%)	0.094	4.367	1.057	0.055	3312.057
9	337.36	HOMO-> LUMO (95%)	0.089	4.344	1.022	0.054	6245.561
10	343.99	HOMO-> LUMO (95%)	0.094	4.272	1.095	0.060	6315.007
11	362.64	HOMO-> LUMO (95%)	0.073	4.036	0.900	0.055	9124.979
12	363.72	HOMO-> LUMO (96%)	0.074	4.030	0.914	0.056	12310.081
13	391.55	HOMO-> LUMO (95%)	0.047	3.680	0.624	0.046	12971.460

**Figure 9:**
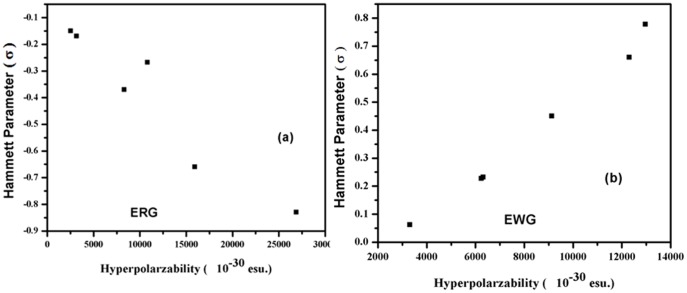
Plot of Hammett parameter with hyperpolarizability (10^−30^ esu) of (a) DMB derivative with electron releasing group and (b) DMB derivates with electron withdrawing groups.

We compare the selected geometry parameters with values of β of DMB derivatives. In molecules, with the releasing groups, the B-C_18_ bond length was observed to be minimum for dimethylamine derivative and maximum for ethyl derivative and follows the order **2**<**3**<**4** = **5**<**6**<**7**, and the B-C_2_ and B-C_10_ bond length slightly decrease from dimethylamine derivative to ethyl derivative. In case of electron withdrawing groups, all the three B-C_18_, B-C_2_ and B-C_10_ bond lengths decreases but the maximum decreases appears in B-C_18_ bond [**8** = **9**>**10**>**11** = **12**>**13**], as substituted phenlyene ring is linked to boron through B-C_18_ bond. Probable reason may be extended- π conjugation improved by electron releasing groups. The well-known resonance and inductive effect [58], [59] may appropriately explain the relationship between donor-acceptor groups and the geometry. The increase in π- conjugation due to +I (inductive) effect of electron releasing groups cause enhance in extent of resonance, results in geometrical modification of derivative on account of attachment of electron releasing substituent. The Hammett constant were correlated with hyperpolarizability. The plots of the β of DMB derivatives verses σ in figure 9 reveals that hyperpolarizability of DMB derivatives with electron releasing group decrease with increasing σ value. While as, in case of DMB derivatives with electron withdrawing groups, the β value increase with increase in Hammett value. The results fit a straight line with a correlation coefficient of 0.92 (or higher) for electron releasing groups, the other one has a correlation coefficient of 0.81 (or lower) for electron withdrawing groups. According to Ulman, inductive and resonance effects have contribution in Hammett substituent constant and both the effects are important to the molecules [58]. The donor group will easily push electrons towards the boron and remain itself slightly electron deficient. This situation affects the magnitude of dipole moments as well as hyperpolarizability. Thus, due to the presence of variety of functional groups (either having electron releasing or electron withdrawing abilities) affect the magnitude of static hyperpolarizability of DMBs.

To highlight the effect of change in bond length and bond angle upon substitution on hyperpolarizability, we plotted the dipole moment and hyperpolarizability of DMB derivatives versus change in key bond lengths and bond angle (see Figure S3 and S4. The observed plots revealed a close correlation between them for electron releasing groups. In DMB derivatives with electron releasing groups, the hyperpolarizability decrease as from **2**>**3**>**4**>**5**>**6**>**7** [26889.28×10^−30^ esu>15924.7×10^−30^ esu>10813.67×10^−30^ esu>8321.161×10^−30^ esu>3156.49×10^−30^ esu>2512.611×10^−30^ esu] and change in bond length (B-C_18_) increases as 0.0004<0.0005<0.0008 = 0.0008<0.0009 respectively. It indicates, that the **2** (dimethylamino group) is the strongest donor group and (methyl group) is the weakest donor group in this system, thus an increase in the strength of donor groups from methyl to dimethylamine have a pronounced impact on the hyperpolarisabilities. In general the trend is similar for other bond lengths for DMB derivatives with electron releasing derivatives. However, there is hardly a correlation between the hyperpolarisabilities and the change in geometrical parameters when the attached groups are electron withdrawing. In case of electron withdrawing groups, the DMB derivative with nitro group show maximum change in geometrical parameters and hyperpolarizability lower than that of fluorine DMB derivatives. We have correlated the Key geometrical parameter BLA (bond length alternation) calculated by the equation 5 with the hyperpolarizability. In case of DMB derivatives with electron donating groups, the hyperpolarizability decreases from **2**>**3**>**4**>**5**>**6**>**7** and the BLA values increases from 0.056(**2**) <0.059(**3**) <0.061(**4**)  = 0.061(**5**) <0.063(**6**) <0.064(**7**). It shows that an increase in the strength of donor groups from methyl to dimethylamino have a clear impact on the β values. Thus, the minimization of the bond length alternation by varying the electron releasing groups is an important manifestation of change in hyperpolarisability. However, there is no good correlation between the hyperpolarizability and the BLA when the substituents are electron withdrawing. The DMB(**12**) and DMB(**13**) have lowest β values, with highest BLA values 0.083 and 0.085 respectively, but in case of **8**, **9**, **10** hyperpolarizability values decreases as **8**>**9**>**10**, but the BLA values does not differ much (0.070(**8**)  = 0.070(**9**) <0.071(**10**). This indicates that the withdrawing groups have little effect on the BLA value and do not contribute to the change of hyperpolarisabilities. It can be concluded that changing the electron donor groups have a better effect on the BLA value and the hyperpolarizability in such electron transport systems.

## Conclusion

In this work, density functional theory has been employed to analyze the electron transport and nonlinear optical properties of DMB derivatives. The geometrical parameters optimized at B3PW91/6-311++G (d, p) level are in agreement with the experimental ones. From the optimized geometries, we have calculated HOMO and LUMO energy levels, electron affinity, reorganization energy, frontiers molecular contours, polarizability and hyperpolarizability in addition to inner-sphere reorganization energies. The absorption energies have been calculated with time-dependent density-functional theory (TDDFT) at the optimized geometries. Replacing the hydrogen of DMB(**1**) ranging from –N(CH_3_)_2_ to NO_2_ groups can cause change in extent of electron transport and nonlinear optical properties of DMB systems. In this study we have correlated the electron transport and nonlinear optical properties with different parameters including geometrical and Hammett parameters. There occurs an enhanced change in dihedral angle due to electron withdrawing substituents, reflecting increased tendency to change in radical-anion as compared to electron releasing groups. A strong correlation was observed between Hammett parameter and the geometrical parameters as the amplitude of the structural modifications increase on moving from electron releasing substituents to electron withdrawing substituents. The effect on reorganization energy was much more prominent in the case of electron withdrawing substituent, with the maximum for fluorine as compared to electron releasing substituents. It was observed that the highest occupied molecular orbitals (HOMOs) of the neutral molecules delocalize primarily over the two mesityl rings, whereas the lowest unoccupied molecular orbitals (LUMOs) localize largely on the B atom and the phenylene of the DMB moiety, with a small contribution from electron releasing or withdrawing substituent. Using two-level model, we observed that for determining the value of hyperpolarizability, the first transition energy is an important factor. In DMB derivatives with electron releasing groups, the hyperpolarizability decrease as from 26889.28×10^−30^ esu>15924.7×10^−30^ esu>10813.67×10^−30^ esu>8321.161×10^−30^ esu>3156.49×10^−30^ esu>2512.611×10^−30^ esu and the key BLA value (B-C_18_) 0.0004<0.0005<0.0008 = 0.0008<0.0009, respectively. We hope that our theoretical study can give some insight to experimentalists for devising a wide variety of materials with extensive electron transport and nonlinear optical properties.

## Supporting Information

Figure S1 **Optimized geometries of the series of studied aryldimesityl borane (DMB) derivates.**
(TIF)

Figure S2 **Plot of geometrical parameters with the Hammett Parameter (a) bond angle versus Hammett parameter (b) bond length versus Hammett parameter, for the series of studied DMB derivates.**
(TIF)

Figure S3 **Correlation plot of bond length with dipole moment of (a) DMB derivatives with electron releasing groups, (b) DMB derivatives with electron withdrawing groups.**
(TIF)

Figure S4 **Correlation plot of bond length with hyperpolarizability of (a) DMB derivatives with electron releasing groups, (b) DMB derivatives with electron withdrawing groups.**
(TIF)

Table S1 **Coordinates of optimized DMB derivatives calculates at B3PW91/6-311++ G (d, p) level.**
(DOCX)

Table S2 **Optimized geometrical parameters (bond length (Å), bond angles (deg.) and dihedral angle (deg.)) of aryldimesityl borane (DMB (1)).**
(DOCX)

## References

[1] Garcia G, Garzon A, Granadino-Roldan JM, Moral M, Navarro A (2011) Fernandez-Gomez, M. Optoelectronic and charge transport properties of oligomers based on phenylethynylene units linked to thioene-acenes: A DFT study. J Phys Chem C 115:6922–5932.

[2] Glogowski ME, Williams JLR (1981) Boron Photochemistry: XV. Determination of the hammett substituent constant for the *p*-dimesitylborylgroup. J Organomet Chem 218: 137-146. (b) Grisdale PJ, Williams JLR, Glogowski ME, Babb BE (1971) Boron photochemistry. Possible role of bridged intermediates in the photolysis of borate complexes. J Org Chem 36:544–549.

[3] Entwistle CD, Marder TB (2004) Applications of three-coordinate organoboron compounds and polymers in optoelectronics, special issue on organic electronics. Chem Mater 16:4574–4585.

[4] Jäkle F (2006) Lewis-Acidic Boron Polymers. Coord Chem Rev 250:1107–1121.

[5] Entwistle CD, Marder TB (2002) Boron Chemistry Lights the Way: Optical Properties of Molecular and Polymeric Systems. Angew Chem Int Ed Engl41: 2927–2931; (2002) Angew Chem 114: 3051–3056. 10.1002/1521-3773(20020816)41:16<2927::AID-ANIE2927>3.0.CO;2-L 12203415

[6] Xue I, ShiLing SUN, NaNa MA, Yong Qing QIU, Qiang FU (2012)Quantum chemical studies on tuning the second-order nonlinear optical molecular switching of triarylborane derivatives. Chin Sci Bull 57:1772–1780.

[7] Parab K, Venkatasubbaiah K, Jäkle F (2006) Luminescent Triarylborane-Functionalized Polystyrene: Synthesis, Photophysical Characterization, and Anion Binding Studies. J Am Chem Soc 128:12879–12885. 17002382 10.1021/ja063302v

[8] Sakuda E, Ando Y, Ito A, Kitamura N (2010) Extremely large dipole moment in the excited singlet state of tris{[p-(*N*dimethylamino) phenylethynyl]duryl}borane. J Phys Chem A 114:9144–9150. 20704330 10.1021/jp1057463

[9] Sun Y, Wang S (2010) Extending II-conjugation of triarylborons with a 2,2- Bpy core: Impact of donor-acceptor geometry on luminescence, anion sensing, and metal ion binding. Inorg Chem 49:4394–4404. 20415452 10.1021/ic1004159

[10] Qin Y, Sukul V, Pagakos D, Cui C, Jäkle F (2005) Preparation of Organoboron Block Copolymers via ATRP of Silicon and Boron Functionalized Monomers. Macromolecules 38:8987–8990.

[11] Qin Y, Cheng G, Sundararaman A, Jäkle F (2002) Well-Defined Boron-Containing Polymeric Lewis Acids. J Am Chem Soc 124:12672–12673. 12392409 10.1021/ja020773i

[12] Matsumi N, Miyata M, Chujo Y (1999)Synthesis of Organoboron p-Conjugated Polymers by Hydroboration Polymerization between Heteroaromatic Diynes and Mesitylborane and Their Light Emitting Properties. Macromolecules 32:4467–4469.

[13] Lam ST, Zhu NY, Yam VM (2009) Synthesis and characterization of luminescent rhenium(I) tricarbonyldiimine complexes with a triarylboron moiety and the study of their fluoride ion-binding properties. Inorg Chem 48:9664–9670. 19746985 10.1021/ic900803a

[14] Zhao Q, LiF Y, Liu SJ, Yu M, Liu Z, et al (2008) HuangC. Highly selective phosphorescent chemosensor for fluoride based on an iridium(III) complex containing arylborane units. Inorg Chem 47:9256–9264. 18811148 10.1021/ic800500c

[15] Sakuda E, Tsuge K, Sasaki Y, Kitamura N (2005) Spectroscopic and excited-state properties of tri-9-anthrylborane III: Crystal and spectroscopic polymorphisms. J Phys Chem B 109:22326–22331. 16853907 10.1021/jp054601u

[16] Liu Y, Xuan WM, ZhangH, Cui Y (2009) Chirality- and threefold-symmetry directed assembly of homochiral octupolar metal-organoboron frameworks. Inorg Chem 48:10018–10023. 19795836 10.1021/ic9002675

[17] Erker G (2005) Tris(pentafluorophenyl) borane: A special boron Lewis acid for special reactions. Dalton Trans 11:1883–1890. 10.1039/b503688g 15909033

[18] Piers WE (2004) The chemistry of perfluoroarylboranes. Adv Organomet Chem 52:1–76.

[19] Chen EYX, Marks TJ (2000) Cocatalysts for metal-catalyzed olefin polymerization: Activators, activation processes, and structure-activity relationships. Chem Rev 100:1391–1434. 11749269 10.1021/cr980462j

[20] Kubo Y, Yamamoto M, Ikeda M, Takeuchi M, Shinkai S, et al (2003) A colorimetric and ratiometric fluorescent chemosensor with three emission changes: Fluoride ion sensing by a triarylborane-porphyrin conjugate. Angew Chem Int Ed 42:2036–2040. 10.1002/anie.200250788 12746816

[21] Yamaguchi S, Akiyama S, Tamao K (2001) Colorimetric fluoride ion sensing by boron-containing π-electron systems. J Am Chem Soc 123:11372–11375. 11707112 10.1021/ja015957w

[22] Shirota Y, Kinoshita M, Noda T, Okumoto K, Ohara T (2000) A novel class of emitting amorphous molecular materials as bipolar radical formants: 2-{4-[Bis(4-methylphenyl)amino]phenyl}- 5-(dimesitylboryl)thiophene and 2-{4-[Bis(9,9-dimethylfluorenyl)amino]phenyl}- 5-(dimesitylboryl)thiophene. J Am Chem Soc 122:11021–11022.

[23] Noda T, Shirota Y (2000) A blue-emitting organic electroluminescent device using a novel emitting amorphous molecular material. J Lumin 87:1168–1170.

[24] Kinoshita M, Kita H, Shirota Y (2002)A novel family of Boron-containing hole-blocking amorphous molecular materials for Blue- and Blue-Violet-Emitting organic electroluminescent devices. AdV Funct Mater 12:780–786.

[25] Doi H, Kinoshita M, Okumoto K, Shirota Y (2003) A novel class of emitting amorphous molecular materials with Bipolar character for electroluminescence. Chem Mater 15:1080–1089.

[26] Uchida M, Ono Y, Yokoi H, Nakano T, Furukawa K (2001) Undoping type of highly efficient organic light emitting diodes. J Photopolym Sci Techno l4:301–305.

[27] Jia WL, Bai DR, McCormick T, Liu QD, Motala M, et al (2004) Three-Coordinate Organoboron Compounds BAr_2_R (Ar = Mesityl, R = 7-Azaindolyl- or 2,2′-Dipyridylamino-Functionalized Aryl or Thienyl) for Electrolumines-cent Devices and Supramolecular Assembly. Chem Eur J 4:994–1006. 10.1002/chem.200305579 14978826

[28] Cheng YC, Silbey RJ, DAda Silva, Filho DA, Calbert JP, et al (2003) Three-Dimensional Band Structure and Bandlike Mobility In Oligoacene Single Crystals: A Theoretical Investigation. J Chem Phys 118:3764.

[29] Lin BC, Cheng CP, Lao ZP (2003) Reorganization Energies In The Transports Of Holes And Electrons In Organic Amines In Organic Electroluminescence Studied By Density Functional Theory. J Phys Chem A107:5241.

[30] Marcus RA (1957) On the Theory Of Oxidation-Reduction Reactions Involving Electron Transfer. II. Applications to Data On The Rates Of Isotopic Exchange Reactions. J Chem Phys 26:867–871.

[31] Marcus RA (1957) On The Theory Of Oxidation-Reduction Reactions Involving Electron Transfer. III. Applications To Data On The Rates Of Organic Redox Reactions. J Chem Phys 26:872–877.

[32] Yamada T, Sato T, Tanaka K, Kaji H (2010)Percolation paths for charge transports in *N,N*′-diphenyl-*N,N*′-di(*m*-tolyl)benzidine (TPD). Org Electron 11:255–265.

[33] Yamada T, Suzuki F, Goto A (2011) Revealing Bipolar Charge-Transport Property Of 4,4′-*N*,*N*′-Dicarbazolylbiphenyl (CBP) By Quantum Chemical Calculations. Org Electron 12:169–178.

[34] Lin BC, Cheng CP, You ZQ, Hsu CP (2005) Charge Transport Properties Of Tris(8-Hydroxyquinolinato)Aluminum(III): Why It Is An Electron Transporter. J Am Chem Soc127:66–67. 10.1021/ja045087t 15631451

[35] Naka S, Okada H, Onnagawa H, Yamaguchi Y, Tsutsui T (2000) Carrier Transport Properties of Organic Materials For EL Device Operation. Synth Met 111–112:331–333.

[36] Yang Y, Geng H, Yin S, Shuai Z, Peng J (2006) First-Principle Band Structure Calculations Of Tris(8-Hydroxyquinolinato)Aluminum. J Phys Chem B 110:3180–3184. 10.1021/jp0540252 16494326

[37] Liu CG, Guan W, Song P, Yan LK, Su ZM (2009) Redox-Switchable Second-Order Nonlinear Optical Responses Of Push-Pull Monotetrathiafulvalene-Metalloporphyrins. Inorg Chem 48:6548–6554. 10.1021/ic9004906 19522472

[38] Dagani R (1996) Devices Based On Electro-Optic Polymers Begin To Enter Marketplace. Chem Eng News 74:22–24.

[39] Lindsay GA, Singer KD (1995) Polymers for Second-Order Nonlinear Optics. Am Chem Soc. Washington. DC

[40] Frisch MJ, Trucks GW, Schlegel HB, Scuseria GE, Robb MA, et al. (2004) GAUSSIAN 03 Revision B 03, Gaussian, Inc., Wallingford CT.

[41] Perdew JP, Burke K, Wang Y (1996) Generalized gradient approximation for the exchange-correlation hole of a many-electron system. Phys Rev B (Condensed Matter) 54:16533–9. 10.1103/physrevb.54.16533 9985776

[42] Binkley JS, Pople JA, Hehre WJ (1980) Self-Consistent Molecular Orbital Small Split-Valence Basis Sets for First-row Elements. J Am Chem Soc 102: 939. (b) McLean AD, Chandler GS (1980) Contracted Gaussian basis sets for molecular calculations. I. Second row atoms, Z = 11−18. J Chem Phys72: 5639. (c) Pandith AH, Islam N, Syed ZF, Rehman S, Bandaru S, Anakuthil A. Density functional theory prediction of geometry and vibrational circular dichroism of bridged triarylamine helicenes (2011) Chem Phys Lett 516:199.

[43] Bauernschmitt R, Ahlrichs R (1996) Treatment of electronic excitations within the adiabatic approximation of time dependent density functional theory. Chem Phys Lett 256:454–464.

[44] Spassova M, Asselberghs I, Verbiest T, Clays K, Botek E, et al (2007) Theoretical investigation on bridged triarylamine helicenes: UV/visible and circular dichroism spectra. Chem Phys Letts 439:213–218.

[45] Zhang R, Du B, Sun G, Sun Y (2010) Experimental and Theoretical Studies On *O*-, *M*- and *P*-Chlorobenzylideneamino antipyrines. Spectrochimica Acta Part A 75:1115–1124. 10.1016/j.saa.2009.12.067 20093073

[46] Sundaraganesan N, Karpagam J, Sebastian S, Cornard JP (2009) The Spectroscopic (FTIR, FT-IR Gas Phase And FT-Raman), First Order Hyperpolarizabilities, NMR Analysis Of 2,4-Dichloroaniline By Ab-Initio HF And Density Functional Methods,. Spectrochimica Acta Part A 73:11–19. 10.1016/j.saa.2009.01.007 19251476

[47] Islam N, Naiz S, Manzoor T, Pandith AH (2014)Theoretical investigations into spectral and non-linear optical properties of brucine and strychnine using density functional theory Spectrochim Acta 131: 461. (b) Islam N, Pandith AH (2014) Analysis of vibrational spectra (FT-IR and VCD) and nonlinear optical properties of [Ru(L)_3_]^2+^ complexes. J Coord Chem 67:3288. 10.1016/j.saa.2014.04.089 24840487

[48] Leffler JE, Grunwald E (1963) Rates and Equilibria of Organic Reactions. Wiley.

[49] Huh JO, Kim H, Lee KM, Lee YS, Do Y, et al (2010) o-Carborane-assisted Lewis acidity enhancement of triarylboranes. Chem Commn 46:1138–1140. 10.1039/b918263b 20126739

[50] Fu Y, Shen W, Li M (2008) Geometries and Electronic Structures of Co-Oligomers and Co-Polymers Based on Tricyclic Nonclassical Thiophene: A Theoretical Study. Macromol Theory Simul 17:385.

[51] Gao H, Zhang H, Zhang H, Gen Y, Su Z-M (2011) Theoretical Study of Isomerism/Phase Dependent Charge Transport Properties in Tris(8-hydroxyquinolinato)aluminum(III). J Phys Chem A 115:9259–9264. 21809889 10.1021/jp202976m

[52] Parisel O, Ellinger Y, Giessner C (1996) The electroaffinity of O_2_ by DFT and coupled MCSCF/peturbation approaches. A computational experiment. Chem Phys Lett 250:178–186.

[53] Ervin KM, Anusiewicz I, Skurski P, Simons J, Lineberger WC (2003) The Only Stable State of O_2_ ^-^ Is the X ^2^Π_g_ Ground State and It (Still!) Has an Adiabatic Electron Detachment Energy of 0.45 eV. J Phys Chem A 107:8521–8529.

[54] Oudar JL, Chemla DS (1977) Hyperpolarisabilities of the nitroanilines and their relations to the excited state dipole moment. J Chem Phys 66:2664–2668.

[55] Kanis DR, Ratner MA, Marks TJ (1992) Calculation and electronic description of quadratic hyperpolarizabilities. Toward a molecular understanding of NLO responses in organotransition metal chromophores. J Am Chem Soc114:10338–10357.

[56] Datta A, Pati SK (2006) Dipolar interactions and hydrogen bonding in supramolecular aggregates: understanding cooperative phenomena for Ist hyperpolarizability. Chem Soc Rev 35:1305–1323. 17225890 10.1039/b605478a

[57] Persoons A (2011) Nonlinear, chirality, magneto-optics: a serendipitous road. Opt Mater Express 1:5–16.

[58] Ulman A (1988) Calculations of dipole moments, optical spectra, and second-order hyperpolarizability coefficients of some mono- and disubstituted stilbene models for the design of nonlinear optical materials. J Phys Chem 92:2385–2390.

[59] Lewis GN (1923) Valence and structure of atoms and molecules. Chemical Catalog Co, New York.

