# Supplementary figures and images for: Electron Transport and Nonlinear Optical Properties of Substituted Aryldimesityl Boranes: A DFT Study
Source: PLoS One. 2014 Dec 5;9(12):e114125. doi: 10.1371/journal.pone.0114125 (PMC4257584; doi:10.1371/journal.pone.0114125)

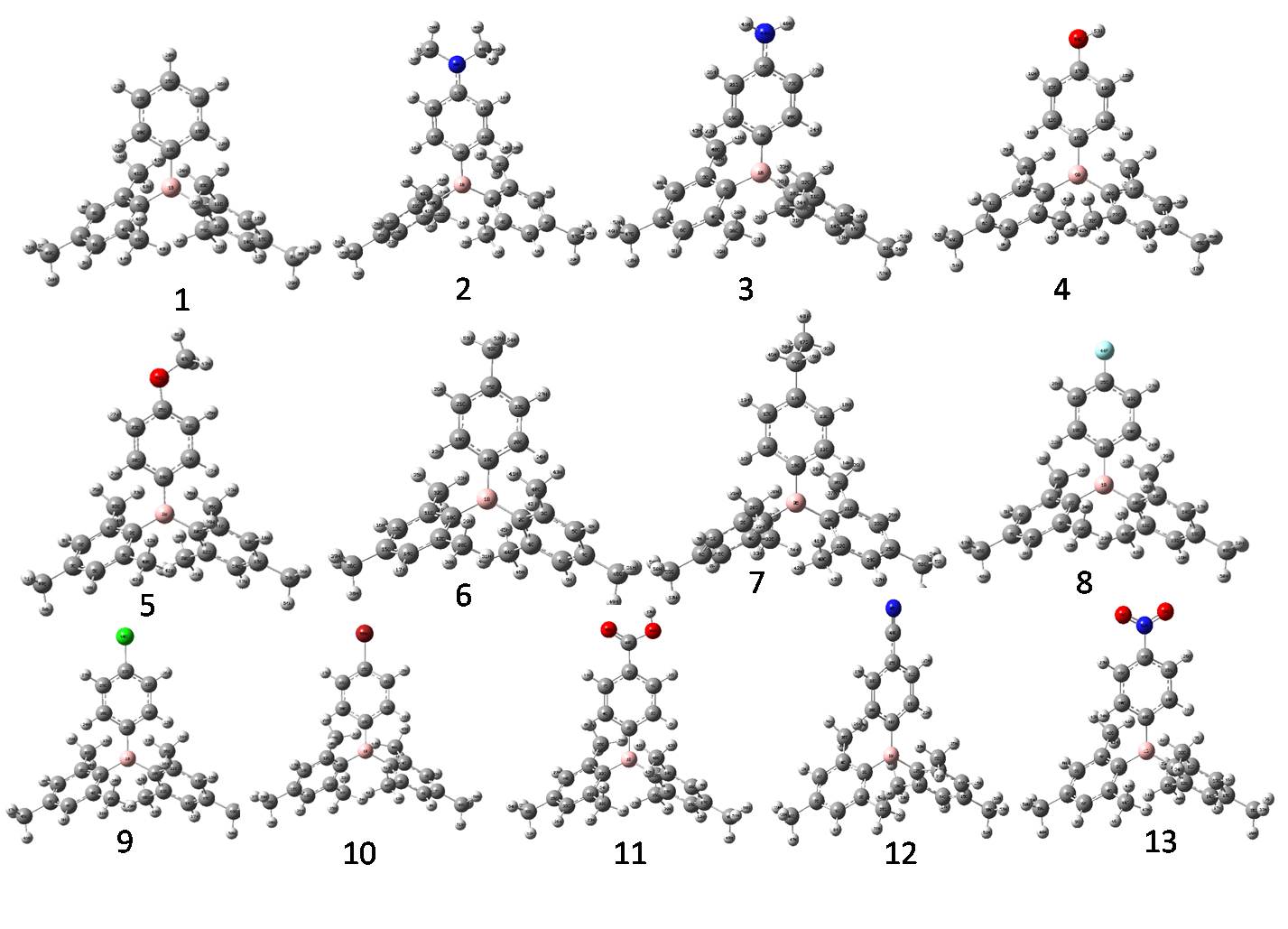

Supplement: Figure S1 — Optimized geometries of the series of studied aryldimesityl borane (DMB) derivates. (TIF) [file pone.0114125.s001.tif]

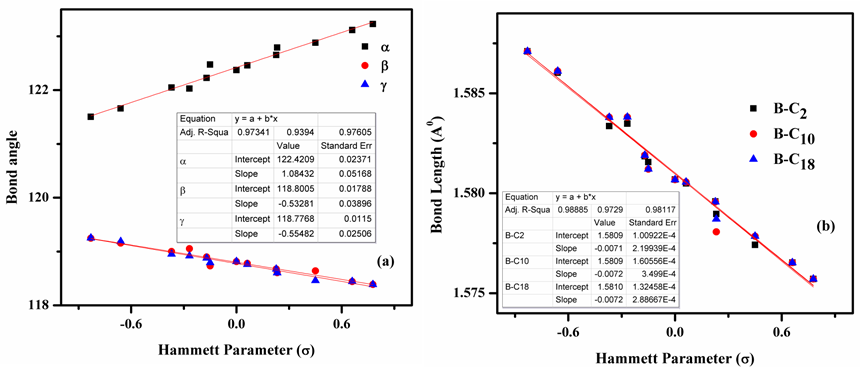

Supplement: Figure S2 — Plot of geometrical parameters with the Hammett Parameter (a) bond angle versus Hammett parameter (b) bond length versus Hammett parameter, for the series of studied DMB derivates. (TIF) [file pone.0114125.s002.tif]

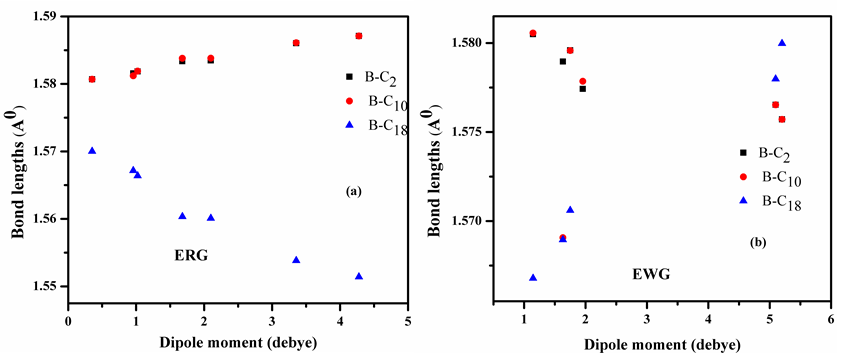

Supplement: Figure S3 — Correlation plot of bond length with dipole moment of (a) DMB derivatives with electron releasing groups, (b) DMB derivatives with electron withdrawing groups. (TIF) [file pone.0114125.s003.tif]

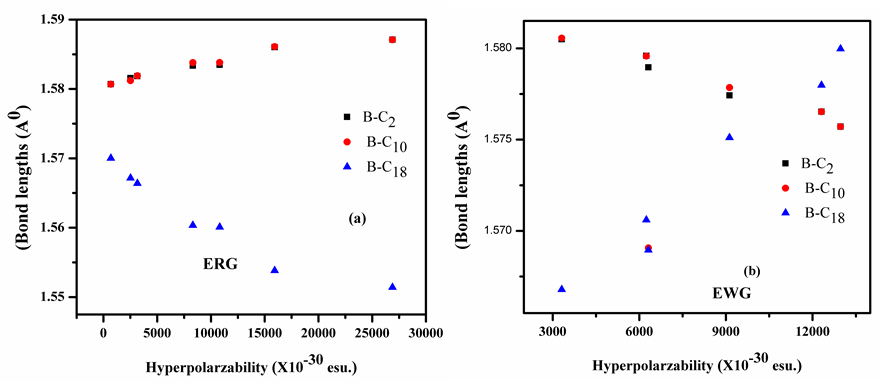

Supplement: Figure S4 — Correlation plot of bond length with hyperpolarizability of (a) DMB derivatives with electron releasing groups, (b) DMB derivatives with electron withdrawing groups. (TIF) [file pone.0114125.s004.tif]
