# Supplementary material for: Electron Transport and Nonlinear Optical Properties of Substituted Aryldimesityl Boranes: A DFT Study
Source: PLoS One. 2014 Dec 5;9(12):e114125. doi: 10.1371/journal.pone.0114125 (PMC4257584; doi:10.1371/journal.pone.0114125)
Supplement: Table S1 — Coordinates of optimized DMB derivatives calculates at B3PW91/6-311++ G (d, p) level. (DOCX) [file pone.0114125.s005.docx]

**Table S1**: Coordinates of optimized DMB derivatives calculates at B3PW91/6-311++ G (d, p) level

**DMB (1)**

Center Atomic Atomic Coordinates (Angstroms)

Number Number Type X Y Z

---------------------------------------------------------------------

1 5 0 0.000018 0.369577 0.000157

2 6 0 -1.384162 -0.392142 0.049338

3 6 0 -2.389291 -0.173734 -0.917699

4 6 0 -1.666031 -1.300016 1.104143

5 6 0 -3.615971 -0.837467 -0.819712

6 6 0 -2.905877 -1.925275 1.175639

7 6 0 -3.900372 -1.711769 0.219661

8 1 0 -4.367802 -0.661777 -1.586032

9 1 0 -3.103229 -2.602540 2.003394

10 6 0 1.384046 -0.392376 -0.049114

11 6 0 2.389311 -0.174107 0.917848

12 6 0 1.665775 -1.300181 -1.104016

13 6 0 3.615994 -0.837776 0.819600

14 6 0 2.905631 -1.925419 -1.175735

15 6 0 3.900282 -1.711943 -0.219929

16 1 0 4.367912 -0.662167 1.585849

17 1 0 3.102844 -2.602650 -2.003550

18 6 0 0.000119 1.939600 0.000104

19 6 0 1.096705 2.670219 -0.492534

20 6 0 -1.096416 2.670362 0.492638

21 6 0 1.093666 4.059332 -0.507777

22 1 0 1.959098 2.131647 -0.873647

23 6 0 -1.093284 4.059479 0.507680

24 1 0 -1.958842 2.131903 0.873836

25 6 0 0.000213 4.756565 -0.000102

26 1 0 1.944966 4.601588 -0.907215

27 1 0 -1.944547 4.601850 0.907041

28 1 0 0.000248 5.842198 -0.000184

29 6 0 0.661965 -1.586746 -2.192365

30 1 0 0.405925 -0.682717 -2.753903

31 1 0 1.061388 -2.311897 -2.904995

32 1 0 -0.269366 -1.987142 -1.784656

33 6 0 2.185768 0.699759 2.132032

34 1 0 1.271816 1.290384 2.083988

35 1 0 2.138889 0.085011 3.038052

36 1 0 3.022427 1.393603 2.258623

37 6 0 5.230362 -2.405667 -0.323966

38 1 0 5.859576 -2.193683 0.543245

39 1 0 5.106951 -3.490997 -0.394175

40 1 0 5.773986 -2.084180 -1.218749

41 6 0 -2.185763 0.700586 -2.131569

42 1 0 -1.270547 1.289317 -2.084478

43 1 0 -2.141567 0.086427 -3.038113

44 1 0 -3.021148 1.396308 -2.256354

45 6 0 -0.662318 -1.586650 2.192560

46 1 0 -0.405474 -0.682472 2.753470

47 1 0 -1.062210 -2.311038 2.905706

48 1 0 0.268641 -1.988067 1.784975

49 6 0 -5.230319 -2.405813 0.323263

50 1 0 -5.106747 -3.491328 0.390459

51 1 0 -5.773041 -2.086761 1.219453

52 1 0 -5.860428 -2.191627 -0.542758

---------------------------------------------------------------------

**DMB (2)**

Center Atomic Atomic Coordinates (Angstroms)

Number Number Type X Y Z

---------------------------------------------------------------------

1 5 0 -0.421325 0.000023 -0.000386

2 6 0 -1.197080 -1.383727 0.047963

3 6 0 -1.014969 -2.374759 -0.940744

4 6 0 -2.086504 -1.674847 1.115281

5 6 0 -1.697686 -3.592680 -0.853026

6 6 0 -2.733428 -2.904316 1.175630

7 6 0 -2.557397 -3.883074 0.196248

8 1 0 -1.548050 -4.332830 -1.636374

9 1 0 -3.397529 -3.105595 2.013283

10 6 0 1.130079 -0.000379 -0.001003

11 6 0 1.880253 -1.131722 0.378856

12 6 0 1.880573 1.130661 -0.381123

13 6 0 3.261208 -1.142028 0.394598

14 1 0 1.352169 -2.031415 0.681218

15 6 0 3.261524 1.140219 -0.397949

16 1 0 1.352746 2.030886 -0.682354

17 6 0 3.996365 -0.001327 -0.002602

18 1 0 3.777522 -2.039371 0.711896

19 1 0 3.778071 2.037747 -0.714351

20 6 0 -1.196494 1.384115 -0.048144

21 6 0 -1.013398 2.374951 0.940541

22 6 0 -2.086560 1.675632 -1.114835

23 6 0 -1.695874 3.593080 0.853482

24 6 0 -2.733191 2.905262 -1.174562

25 6 0 -2.556249 3.883830 -0.195129

26 1 0 -1.545466 4.333077 1.636825

27 1 0 -3.397806 3.106869 -2.011728

28 6 0 -0.151873 -2.170407 -2.163277

29 1 0 0.437047 -1.255514 -2.117546

30 1 0 0.543201 -3.005976 -2.293394

31 1 0 -0.773116 -2.127084 -3.065031

32 6 0 -2.332388 0.689520 -2.228551

33 1 0 -1.410198 0.455733 -2.769734

34 1 0 -2.730395 -0.254378 -1.847759

35 1 0 -3.044661 1.092231 -2.952495

36 6 0 -2.331345 -0.688577 2.229081

37 1 0 -1.408828 -0.455381 2.769970

38 1 0 -2.728897 0.255571 1.848450

39 1 0 -3.043618 -1.090867 2.953258

40 6 0 -0.149365 2.170269 2.162361

41 1 0 -0.769775 2.128222 3.064745

42 1 0 0.438403 1.254632 2.116585

43 1 0 0.546869 3.005054 2.291303

44 7 0 5.366400 -0.002178 -0.004465

45 6 0 6.095043 -1.180696 0.413301

46 1 0 7.164032 -0.985078 0.338286

47 1 0 5.874071 -1.451454 1.453531

48 1 0 5.866363 -2.047288 -0.219423

49 6 0 6.095409 1.181202 -0.407583

50 1 0 7.164338 0.981117 -0.344427

51 1 0 5.867531 1.468041 -1.441715

52 1 0 5.873993 2.039104 0.239851

53 6 0 -3.274381 5.202430 -0.283196

54 1 0 -2.974223 5.757963 -1.178084

55 1 0 -4.358461 5.060639 -0.340422

56 1 0 -3.063409 5.829628 0.585911

57 6 0 -3.275641 -5.201563 0.285065

58 1 0 -2.973998 -5.757503 1.179207

59 1 0 -4.359588 -5.059600 0.344264

60 1 0 -3.066297 -5.828467 -0.584645

---------------------------------------------------------------------

**DMB (3)**

Center Atomic Atomic Coordinates (Angstroms)

Number Number Type X Y Z

---------------------------------------------------------------------

1 5 0 0.000169 0.115072 0.000356

2 6 0 -1.381914 -0.660698 0.059784

3 6 0 -2.382740 -0.473703 -0.918196

4 6 0 -1.662298 -1.553665 1.127003

5 6 0 -3.600750 -1.154548 -0.819557

6 6 0 -2.892375 -2.198357 1.198357

7 6 0 -3.881386 -2.016943 0.230269

8 1 0 -4.348880 -1.001470 -1.594571

9 1 0 -3.085747 -2.865119 2.035736

10 6 0 1.385343 -0.655321 -0.058965

11 6 0 2.385225 -0.465848 0.919385

12 6 0 1.669091 -1.546408 -1.126859

13 6 0 3.605502 -1.142587 0.820897

14 6 0 2.901275 -2.187118 -1.198072

15 6 0 3.889284 -2.003380 -0.229430

16 1 0 4.352844 -0.987677 1.596325

17 1 0 3.097178 -2.852523 -2.035928

18 6 0 -0.003354 1.668884 -0.000787

19 6 0 -1.131814 2.412420 0.401829

20 6 0 1.121212 2.417172 -0.405489

21 6 0 -1.140882 3.793997 0.415791

22 1 0 -2.022472 1.879619 0.721489

23 6 0 1.123033 3.798795 -0.423237

24 1 0 2.015345 1.888218 -0.721834

25 6 0 -0.010485 4.516367 -0.003871

26 1 0 -2.026038 4.332578 0.743510

27 1 0 2.002519 4.341159 -0.759848

28 6 0 0.672316 -1.799938 -2.229370

29 1 0 -0.266058 -2.200088 -1.837406

30 1 0 0.429341 -0.880891 -2.771887

31 1 0 1.070322 -2.513382 -2.954674

32 6 0 2.187884 0.397962 2.142548

33 1 0 1.268021 0.979852 2.106440

34 1 0 2.158933 -0.222220 3.045563

35 1 0 3.019598 1.099424 2.262678

36 6 0 -0.664272 -1.804979 2.228929

37 1 0 0.275906 -2.200035 1.836149

38 1 0 -0.425513 -0.885933 2.773381

39 1 0 -1.059024 -2.521716 2.952775

40 6 0 -2.189360 0.391868 -2.140733

41 1 0 -1.267646 0.970953 -2.109235

42 1 0 -2.167897 -0.226801 -3.044957

43 1 0 -3.019123 1.096602 -2.254782

44 7 0 -0.031291 5.891998 -0.051312

45 1 0 -0.726584 6.360845 0.506106

46 1 0 0.857193 6.364793 -0.084358

47 6 0 -5.199906 -2.733428 0.330938

48 1 0 -5.059159 -3.818193 0.376806

49 1 0 -5.741809 -2.440025 1.236305

50 1 0 -5.839098 -2.513935 -0.527207

51 6 0 5.210214 -2.715383 -0.330309

52 1 0 5.072886 -3.800314 -0.381792

53 1 0 5.753392 -2.415927 -1.232937

54 1 0 5.846707 -2.498091 0.530414

---------------------------------------------------------------------

**DMB (4)**

Center Atomic Atomic Coordinates (Angstroms)

Number Number Type X Y Z

---------------------------------------------------------------------

1 6 0 -3.626099 -1.075679 0.821148

2 6 0 -2.394318 -0.420471 0.917673

3 6 0 -1.395225 -0.637791 -0.055690

4 6 0 -1.689841 -1.535137 -1.115719

5 6 0 -2.933633 -2.152722 -1.185456

6 6 0 -3.921839 -1.940453 -0.222753

7 1 0 -4.372819 -0.900497 1.592677

8 1 0 -3.139103 -2.822980 -2.016979

9 5 0 -0.000848 0.110442 -0.002062

10 6 0 0.021088 1.670632 -0.004858

11 6 0 1.149461 2.392592 0.425498

12 6 0 -1.086917 2.428196 -0.436725

13 6 0 1.172618 3.778213 0.440792

14 1 0 2.026005 1.847856 0.762593

15 6 0 -1.073709 3.811324 -0.454568

16 1 0 -1.977801 1.907441 -0.774229

17 6 0 0.059648 4.494429 -0.006825

18 1 0 2.053365 4.310622 0.792904

19 1 0 -1.925449 4.385292 -0.802244

20 6 0 1.372995 -0.675522 0.054803

21 6 0 2.376905 -0.491516 -0.920749

22 6 0 1.644765 -1.572744 1.120962

23 6 0 3.591173 -1.178124 -0.820041

24 6 0 2.871871 -2.222622 1.194300

25 6 0 3.864578 -2.043453 0.229474

26 1 0 4.341790 -1.028297 -1.593159

27 1 0 3.060004 -2.892106 2.030597

28 6 0 2.186882 0.371894 -2.145075

29 1 0 2.141219 -0.251003 -3.045471

30 1 0 1.277150 0.969481 -2.107006

31 1 0 3.029711 1.058246 -2.273602

32 6 0 0.642181 -1.822283 2.219248

33 1 0 0.412107 -0.905473 2.771278

34 1 0 -0.301375 -2.204279 1.822011

35 1 0 1.028434 -2.548734 2.937840

36 6 0 -2.184030 0.449146 2.134437

37 1 0 -2.185188 -0.162918 3.043322

38 1 0 -1.245975 1.001697 2.105640

39 1 0 -2.993954 1.178049 2.238114

40 6 0 -0.693229 -1.819805 -2.210898

41 1 0 -0.431325 -0.912332 -2.764014

42 1 0 0.235961 -2.232700 -1.810494

43 1 0 -1.101486 -2.534399 -2.929237

44 8 0 0.024343 5.848634 -0.028457

45 6 0 5.179433 -2.765949 0.332918

46 1 0 5.720029 -2.475620 1.239993

47 1 0 5.033322 -3.849970 0.377897

48 1 0 5.821872 -2.548914 -0.523314

49 6 0 -5.256752 -2.625416 -0.324201

50 1 0 -5.804517 -2.292973 -1.212464

51 1 0 -5.140478 -3.710854 -0.404513

52 1 0 -5.879032 -2.417261 0.548981

53 1 0 0.858998 6.190899 0.304676

---------------------------------------------------------------------

**DMB (5)**

Center Atomic Atomic Coordinates (Angstroms)

Number Number Type X Y Z

---------------------------------------------------------------------

1 5 0 -0.177213 -0.000843 -0.012209

2 6 0 -1.302054 -1.114726 -0.049528

3 6 0 -2.269929 -1.122020 -1.088500

4 6 0 -1.365424 -2.144091 0.914634

5 6 0 -3.225978 -2.129732 -1.147620

6 6 0 -2.354463 -3.129350 0.829213

7 6 0 -3.291121 -3.147078 -0.194078

8 1 0 -3.945513 -2.122641 -1.963071

9 1 0 -2.387959 -3.903470 1.592816

10 6 0 -0.525565 1.542786 0.053421

11 6 0 -0.067940 2.451408 -0.925927

12 6 0 -1.290535 2.062229 1.130427

13 6 0 -0.367744 3.813299 -0.817576

14 6 0 -1.552008 3.425508 1.210769

15 6 0 -1.102342 4.324788 0.242412

16 1 0 -0.013941 4.489681 -1.592735

17 1 0 -2.125996 3.799685 2.055480

18 6 0 1.320752 -0.435486 -0.044355

19 6 0 2.347370 0.424402 0.379218

20 6 0 1.716277 -1.711475 -0.500401

21 6 0 3.685249 0.050047 0.369429

22 1 0 2.087675 1.417143 0.734450

23 6 0 3.041522 -2.095578 -0.544936

24 1 0 0.954558 -2.408822 -0.835506

25 6 0 4.039427 -1.217101 -0.102877

26 1 0 4.438575 0.744423 0.720952

27 1 0 3.341029 -3.071584 -0.910597

28 6 0 -1.811384 1.173099 2.231481

29 1 0 -2.477831 0.400071 1.840855

30 1 0 -0.998685 0.661919 2.756962

31 1 0 -2.363635 1.757373 2.970991

32 6 0 -0.444766 -2.210610 2.110096

33 1 0 0.360022 -1.478099 2.067812

34 1 0 -1.008690 -2.037810 3.033655

35 1 0 0.013494 -3.200961 2.195082

36 6 0 0.686351 2.020805 -2.161146

37 1 0 1.581444 2.633917 -2.305015

38 1 0 1.001849 0.979165 -2.124222

39 1 0 0.061667 2.151698 -3.051999

40 6 0 -2.275789 -0.074694 -2.173536

41 1 0 -2.393630 0.930199 -1.760722

42 1 0 -1.342963 -0.080998 -2.746041

43 1 0 -3.092592 -0.251883 -2.876891

44 8 0 5.306861 -1.683800 -0.171384

45 6 0 6.359900 -0.841614 0.259127

46 1 0 7.275737 -1.413666 0.116659

47 1 0 6.410363 0.075539 -0.338602

48 1 0 6.257987 -0.581270 1.318752

49 6 0 -4.344258 -4.217099 -0.280969

50 1 0 -5.350070 -3.785977 -0.243286

51 1 0 -4.266818 -4.772818 -1.221211

52 1 0 -4.255450 -4.932706 0.539461

53 6 0 -1.412098 5.792389 0.351779

54 1 0 -2.492679 5.969233 0.353928

55 1 0 -1.015004 6.212759 1.281537

56 1 0 -0.982456 6.353878 -0.480715

---------------------------------------------------------------------

**DMB (6)**

Center Atomic Atomic Coordinates (Angstroms)

Number Number Type X Y Z

---------------------------------------------------------------------

1 5 0 -0.000121 0.090490 -0.001645

2 6 0 1.381181 -0.678845 -0.051922

3 6 0 2.385887 -0.469252 0.917435

4 6 0 1.660093 -1.585620 -1.108297

5 6 0 3.608623 -1.140695 0.820395

6 6 0 2.895849 -2.219092 -1.178761

7 6 0 3.889668 -2.014534 -0.220157

8 1 0 4.360054 -0.971379 1.588604

9 1 0 3.090341 -2.895678 -2.007797

10 6 0 -1.386952 -0.668556 0.051297

11 6 0 -2.390341 -0.454949 -0.918586

12 6 0 -1.672240 -1.569563 1.110891

13 6 0 -3.618091 -1.116741 -0.818745

14 6 0 -2.912708 -2.193500 1.183923

15 6 0 -3.905343 -1.984740 0.225018

16 1 0 -4.368477 -0.944434 -1.587300

17 1 0 -3.111980 -2.865799 2.015303

18 6 0 0.005917 1.656872 -0.003280

19 6 0 -1.094083 2.398783 0.464552

20 6 0 1.109746 2.388529 -0.476360

21 6 0 -1.084606 3.785815 0.474226

22 1 0 -1.969064 1.869375 0.829803

23 6 0 1.109123 3.776173 -0.494259

24 1 0 1.978385 1.851488 -0.845622

25 6 0 0.015955 4.500178 -0.009810

26 1 0 -1.946407 4.328542 0.853350

27 1 0 1.970647 4.311029 -0.884791

28 6 0 -0.670674 -1.850530 2.202681

29 1 0 -0.413278 -0.942913 2.757733

30 1 0 -1.072573 -2.569642 2.920073

31 1 0 0.260252 -2.256022 1.798973

32 6 0 -2.184675 0.412850 -2.136907

33 1 0 -1.269861 1.002206 -2.090467

34 1 0 -2.138439 -0.206061 -3.040136

35 1 0 -3.020268 1.107407 -2.267052

36 6 0 -5.236239 -2.676853 0.330273

37 1 0 -5.864175 -2.467747 -0.538596

38 1 0 -5.113976 -3.762055 0.404798

39 1 0 -5.780812 -2.351456 1.223078

40 6 0 2.187023 0.405081 2.132245

41 1 0 1.272717 0.995278 2.087075

42 1 0 2.144616 -0.209060 3.038882

43 1 0 3.023947 1.099509 2.254563

44 6 0 0.656934 -1.862322 -2.199678

45 1 0 0.409394 -0.954962 -2.759645

46 1 0 1.052357 -2.589088 -2.912938

47 1 0 -0.278456 -2.256272 -1.794900

48 6 0 5.215088 -2.717561 -0.322087

49 1 0 5.084515 -3.802544 -0.385204

50 1 0 5.759557 -2.405394 -1.219642

51 1 0 5.846959 -2.504436 0.542942

52 6 0 0.031673 6.002787 0.015281

53 1 0 0.381177 6.370585 0.986911

54 1 0 0.699591 6.409063 -0.747820

55 1 0 -0.966992 6.415735 -0.148168

---------------------------------------------------------------------

**DMB (7)**

Center Atomic Atomic Coordinates (Angstroms)

Number Number Type X Y Z

---------------------------------------------------------------------

1 6 0 -2.062598 -3.345471 -0.775409

2 6 0 -1.211049 -2.246747 -0.926577

3 6 0 -1.187297 -1.226399 0.049549

4 6 0 -2.053434 -1.358780 1.166465

5 6 0 -2.872049 -2.476248 1.288072

6 6 0 -2.896622 -3.485894 0.324735

7 1 0 -2.069064 -4.111778 -1.547490

8 1 0 -3.512594 -2.563794 2.162581

9 5 0 -0.200675 0.004924 -0.053651

10 6 0 1.339170 -0.268098 -0.155218

11 6 0 2.223006 0.689970 -0.684036

12 6 0 1.901381 -1.483630 0.275138

13 6 0 3.584651 0.443576 -0.787461

14 1 0 1.824855 1.640165 -1.027483

15 6 0 3.266496 -1.719885 0.198242

16 1 0 1.248462 -2.252063 0.678433

17 6 0 4.132126 -0.762387 -0.339389

18 1 0 4.238660 1.196478 -1.219733

19 1 0 3.671496 -2.665726 0.548893

20 6 0 -0.709018 1.502564 -0.055331

21 6 0 -0.251175 2.446429 0.890332

22 6 0 -1.617583 1.950048 -1.050168

23 6 0 -0.686259 3.773776 0.826908

24 6 0 -2.011603 3.283042 -1.089119

25 6 0 -1.559238 4.217561 -0.156191

26 1 0 -0.327805 4.477636 1.574875

27 1 0 -2.693710 3.604375 -1.872951

28 6 0 -0.404482 -2.182931 -2.201224

29 1 0 -1.060845 -1.979247 -3.055013

30 1 0 0.363316 -1.410516 -2.180674

31 1 0 0.090309 -3.138694 -2.398476

32 6 0 -2.089892 -0.329025 2.267776

33 1 0 -1.120172 -0.237788 2.767319

34 1 0 -2.348596 0.660663 1.883405

35 1 0 -2.825140 -0.602722 3.027790

36 6 0 0.645427 2.080879 2.048563

37 1 0 0.079887 2.102279 2.987202

38 1 0 1.086608 1.090147 1.948449

39 1 0 1.463586 2.800207 2.150969

40 6 0 -2.151474 1.021800 -2.112194

41 1 0 -1.346821 0.604439 -2.725672

42 1 0 -2.693053 0.179530 -1.674663

43 1 0 -2.831846 1.552823 -2.781725

44 6 0 5.617640 -1.007518 -0.396271

45 1 0 6.038350 -0.491998 -1.266174

46 1 0 5.804049 -2.076493 -0.544955

47 6 0 6.340296 -0.537823 0.871744

48 1 0 7.414923 -0.730502 0.803479

49 1 0 6.197221 0.534758 1.029922

50 1 0 5.957903 -1.056559 1.755281

51 6 0 -2.006533 5.651647 -0.224358

52 1 0 -1.687593 6.120594 -1.161049

53 1 0 -3.097614 5.728907 -0.180576

54 1 0 -1.594897 6.237626 0.600141

55 6 0 -3.802407 -4.676094 0.480318

56 1 0 -3.603162 -5.205697 1.417473

57 1 0 -4.854249 -4.372288 0.499293

58 1 0 -3.673980 -5.385540 -0.339997

---------------------------------------------------------------------
