# Supplementary material for: Electron Transport and Nonlinear Optical Properties of Substituted Aryldimesityl Boranes: A DFT Study
Source: PLoS One. 2014 Dec 5;9(12):e114125. doi: 10.1371/journal.pone.0114125 (PMC4257584; doi:10.1371/journal.pone.0114125)
Supplement: Table S2 — Optimized geometrical parameters (bond length (Å), bond angles (deg.) and dihedral angle (deg.)) of aryldimesityl borane (DMB (1)). (DOCX) [file pone.0114125.s006.docx]

**Table S2**: Optimized geometrical parameters (bond length (Å), bond angles (deg.) and dihedral angle (deg.)) of aryldimesitylborane (DMB (1)).

| Bond Length | Theoretical (Å) | Exp.  (Å) | Bond and Dihedral Angles | Theoretical (degrees) | Exp. (degrees) |
| --- | --- | --- | --- | --- | --- |
| B-C_18_ | 1.570 | 1.582 | C_18_BC_10_ | 118.82 | 118.40 |
| C_18_-C_19_ | 1.407 | 1.401 | C_2_BC_10_ | 122.37 | 122.61 |
| C_19_-C_21_ | 1.389 | 1.383 | C_18_BC_2_ | 118.82 | 119.91 |
| C_21_-C_25_ | 1.393 | 1.393 | C_33_C_11_C_10_B | 5.54 | 5.65 |
| C_25_-C_23_ | 1.393 | 1.396 | C_29_C_12_C_10_B | 0.80 | 0.78 |
| C_23_-C_20_ | 1.389 | 1.385 | C_2_C_4_C_45_B | 0.97 | 0.88 |
| C_20_-C_18_ | 1.407 | 1.398 | C_41_C_2_C_3_B | 1.51 | 1.89 |
| B-C_10_ | 1.581 | 1.578 | C_11_C_10_BC_18_ | 56.68 | 56.65 |
| C_33_-C_11_ | 1.510 | 1.509 | C_4_C_2_BC_18_ | 121.58 | 121.58 |
| C_12_-C_29_ | 1.508 | 1.610 | C_12_C_10_BC_18_ | 121.56 | 121.57 |
| C_15_-C_37_ | 1.504 | 1.509 | C_3_C_2_BC_18_ | 56.67 | 56.67 |
| B-C_2_ | 1.581 | 1.584 | C_12_C_10_BC_2_ | 58.43 | 58.41 |
| C_3_-C_41_ | 1.510 | 1.507 | C_4_C_2_BC_10_ | 58.43 | 58.43 |
| C_4_-C_45_ | 1.508 | 1.508 | C_20_C_18_BC_2_ | 22.15 | 22.16 |
| C_7_-C_49_ | 1.504 | 1.509 | C_19_C_18_BC_10_ | 22.15 | 22.15 |
